# Diversified secondary metabolite biosynthesis gene repertoire revealed in symbiotic dinoflagellates

**DOI:** 10.1038/s41598-018-37792-0

**Published:** 2019-02-04

**Authors:** Girish Beedessee, Kanako Hisata, Michael C. Roy, Frances M. Van Dolah, Noriyuki Satoh, Eiichi Shoguchi

**Affiliations:** 1 Marine Genomics Unit, Okinawa Institute of Science and Technology Graduate University, Onna, Okinawa, 904-0495 Japan; 2 Instrumental Analysis Section, Okinawa Institute of Science and Technology Graduate University, Onna, Okinawa, 904-0495 Japan; 3 College of Charleston, School of Sciences and Mathematics, 66 George St., Charleston, South Carolina 29424 USA

## Abstract

Symbiodiniaceae dinoflagellates possess smaller nuclear genomes than other dinoflagellates and produce structurally specialized, biologically active, secondary metabolites. Till date, little is known about the evolution of secondary metabolism in dinoflagellates as comparative genomic approaches have been hampered by their large genome sizes. Here, we overcome this challenge by combining genomic and metabolomics approaches to investigate how chemical diversity arises in three decoded Symbiodiniaceae genomes (clades A3, B1 and C). Our analyses identify extensive diversification of polyketide synthase and non-ribosomal peptide synthetase genes from two newly decoded genomes of *Symbiodinium tridacnidorum* (A3) and *Cladocopium* sp. (C). Phylogenetic analyses indicate that almost all the gene families are derived from lineage-specific gene duplications in all three clades, suggesting divergence for environmental adaptation. Few metabolic pathways are conserved among the three clades and we detect metabolic similarity only in the recently diverged clades, B1 and C. We establish that secondary metabolism protein architecture guides substrate specificity and that gene duplication and domain shuffling have resulted in diversification of secondary metabolism genes.

## Introduction

Dinoflagellates of the family Symbiodiniaceae^1^ (previously known as the genus *Symbiodinium*) exist freely in symbiotic associations with many invertebrates, such as corals, clams, and anemones. This invertebrate-Symbiodiniaceae mutualism seems to provide a competitive advantage^2^, resulting in the production and exchange of metabolites by both organisms^3^. Members of this family are sources of unusual, large, polyhydroxyl and polyether compounds or so-called “super-carbon-chain compounds (SCCs),” composed of long-chain backbones functionalized by oxygen^4^. The Symbiodiniaceae SCCs are polyketide metabolites, that are biosynthesized via an assembly line mechanism by two important classes of modular enzymes, polyketide synthase (PKS) and non-ribosomal peptide synthase (NRPS)^5^. PKSs comprise three core domains: an acyl-transferase (AT) domain, an acyl-carrier protein (ACP), and a ketosynthase (KS) domain that work with optional domains^6^. Polyketide synthases are also closely related to fatty acid synthases (FASs) and share the same core of enzymatic activities, implying a common evolutionary history^7^. Based on protein organization, PKSs are further categorized into three types (Type I, II and III), and FASs into two (Type I and II)^8^.

On the other hand, NRPSs are modular multi-enzyme complexes that synthesize a diverse array of biological active peptides or lipopeptides^9^. Biosynthesis of non-ribosomal peptides occurs via the action of catalytic modules within NRPS that are composed of three compulsory domains: adenylation (A-domain), thiolation (T-domain), and condensation (C-domain), supported by other domains^10^. PKS and NRPS pathways often cross-talk such that a polyketide product is elongated by NRPS or *vice versa* to produce hybrid natural products, thereby increasing structural diversity^11^. Pathways involved in secondary metabolite biosynthesis are among the most rapidly evolving genetic elements^12^. Mutations, domain rearrangements, and module duplications within *PKS* and *NRPS* genes account for generation of novel, diverse small-molecules^12^. Thus, there exist several entry points where combinatorial potential can arise.

Several of these SCCs such as zooxanthellatoxins (ZTs) and zooxanthellamides (ZADs) have been isolated from several Symbiodiniaceae clades and a clade-to-metabolite relationship has been proposed and experimentally supported, in which strains of specific Symbiodiniaceae clades produce specific metabolites^13^. Nakamura *et al*. (ref.^14^) suggested the existence of shared biogenetic processes, such as the polyketide pathway with glycine as the starting substrate, yielding products with structural similarities to palytoxins and ZTs. Over the years, other secondary metabolites have been isolated from these clades, but their ecological roles and biosynthetic pathways have yet to be identified^15^. A preliminary genomic survey reported the presence and organization of secondary metabolite genes in *Breviolum minutum* (B1), overcoming limitations of previous transcriptomic surveys^16^. Availability of new Symbiodiniaceae genomes now allows us to survey and compare genes associated with metabolite biosynthesis^17–20^. However, how chemical diversity arises within Symbiodiniaceae is still unknown. Evolution of novel chemistry depends on diversity-generating metabolism, which comprises broad-substrate enzymes^21^. Metabolic pathways accept many different substrates, generating diverse chemical products and this provides organisms with unique chemistry to face environmental challenges^22^.

To investigate existence of shared biosynthetic pathways, we cultured three species of the family Symbiodiniaceae namely *Symbiodinium tridacnidorum* (a.k.a clade A3), *Breviolum minutum* (a.k.a clade B1), and *Cladocopium* sp. (a.k.a clade C) that produce different metabolites, and surveyed their genomes^17,20^ for genes involved in polyketide and non-ribosomal peptide biosynthesis. Additionally, we examined how these genomes are equipped to expand their gene repertoire for biosynthesis of complex secondary metabolites and suggest possible diversification mechanisms that may contribute to such chemical variability and modularity.

## Results

### Phylogenetic and syntenic analyses of ketosynthase and acyltransferase domains

**Figure 1:**
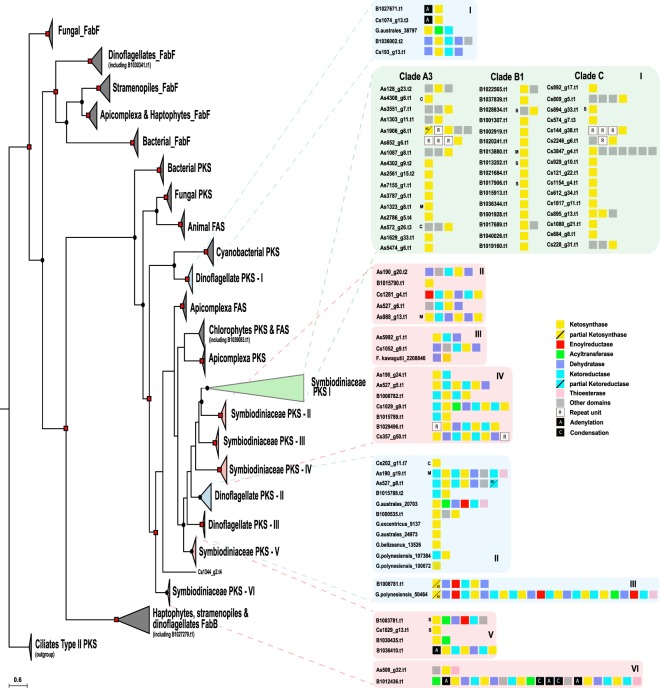
Phylogenetic tree of ketosynthase (KS) domains of prokaryotic and eukaryotic polyketide and fatty acid synthases. Analysis of ketosynthase, FabB-KASI, and FabF-KASII domains shows diversification of these domains into nine groups, comprised of mono-and multifunctional domains. Dots and squares indicate posterior probabilities of 0.70–0.89 and 0.9–1.0, respectively, generated by Bayesian inference. Inserts provide details of sub-groups as well as gene model architecture. C, M, and S denote chloroplasts, mitochondria, and secretory signal peptides, respectively.

The tree shows that majority KS domains clustered according to their domain organization types under a reliable node (BI posterior probability: 0.79 & maximum likelihood probability: 99) (Fig. 1, Supplemental Information, Figs S1 and S2). Recently, Kohli *et al*. (ref.^23^) described contigs encoding multiple PKS domains in the dinoflagellates, *Gambierdiscus excentricus* and *Gambierdiscus polynesiensis*. Those sequences clustered into three dinoflagellate groups (Dinoflagellate PKS I, II and III) (blue highlighted inset of Fig. 1). We confirmed the presence of 25 KS sequences each from clades A3 and C. Our analysis showed only one gene model (B1030341.t1) associated with Type II fatty acid synthesis (FabF-KASII) and one gene model (B1027279.t1) in the FabB-KASI group. The result mirrored the clear demarcation between Type II FAS and Type I PKS & FAS^24^. Our analysis additionally revealed the expanded nature of *KS* genes into nine PKS groups (Dinoflagellate PKS I–III and Symbiodiniaceae PKS I–VI) associated with either mono- or multifunctional domains (Fig. 1). One group (Dinoflagellate PKS-I) was closely related to cyanobacterial KS sequences. Scanning the GC profile of PKS-I group scaffolds of clade C showed some regions of higher GC content (45–46.5%), compared to the average genomic GC content of 43.0%, indicative of gene transfer (Supplemental Information, Fig. S3). ~3% (3/83) of the sequences contain the cTP (chloroplast transit peptide) signal while 12% (10/83) contained mitochondrial targeting peptide (mTP) or secretory signal each (Fig. 1).

**Figure 2:**
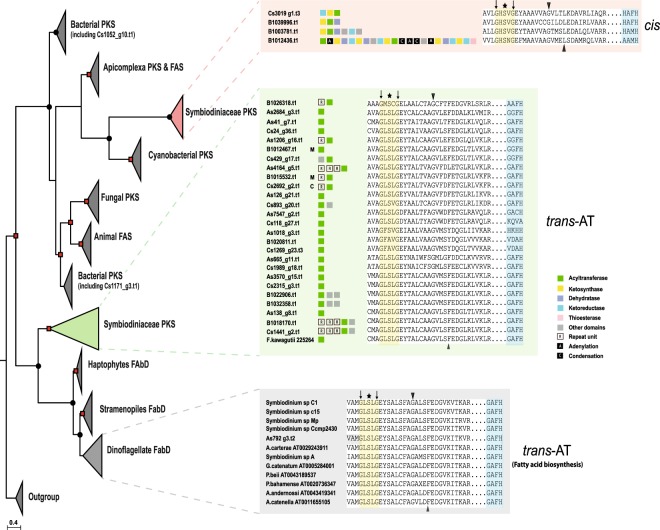
Phylogenetic tree of acyltransferase (AT) domain of prokaryotic and eukaryotic polyketide and fatty acid synthases. Analysis of acyltransferase domain show clear demarcation between *cis-* and *trans*-AT. Dots and square indicate posterior probability 0.70–0.89 and 0.9–1.0, respectively, generated by Bayesian inference. Details of sequences are provided in box inserts. Asterisk indicates active site residue, black triangles indicate conserved residues characteristic for specific substrate groups, and black arrows indicate overall conserved residues used by HMM^25^. The motif, GAFH, present in most Symbiodiniaceae sequences reflects the prediction of I-TASSER. C, M and S depicts chloroplast, mitochondria, and secretory signal peptide, respectively.

A striking feature among the three genomes is the high number (26) of *trans*-AT genes in contrast to *cis*-AT (4) (Fig. 2). A phylogenetic tree of the AT domain consisted of two main nodes, *cis*-AT and *trans*-AT (BI posterior probability: 1.00 & maximum likelihood probability: 81) (Fig. 2, Supplemental Information, Figs S4 and S5), that deviated from the classical substrate-based clustering^25^. Alignment of the *trans*-AT motif revealed a deviation from the usual GHSxG conserved motif to GLSxG where x can be any residue; thus, a change from a basic amino acid (histidine) to an aliphatic one (leucine) while *cis*-AT maintained their GHSxG motif (Fig. 2). Protein structure and function prediction by I-TASSER showed that most Symbiodiniaceae AT sequences pertain to the family of malonyl-CoA ACP transferase, based on the motif GAFH (highlighted blue in Fig. 2). Downstream of the active site serine, a motif (YASH or HAFH) is involved in the choice of either methylmalonyl-CoA or malonyl-CoA, respectively^26^. ~9% (3/33) of AT gene models contained the cTP or mTP signals (Fig. 2).

Comparative visualization of *PKS*-containing scaffolds from the three genomes showed extensive duplication events in the three clades between genes associated with polyketide biosynthetic clusters (Supplemental Information, Fig. S6a). Genomic synteny was observed between clades B1 and A3 (8 syntenic blocks), clades B1 and C (10 syntenic blocks) and clades A3 and C (7 syntenic blocks) (Supplemental Information, Fig. S6b–d) while only four *PKS*-containing gene clusters were found to be shared among all three clades (green boxes in Supplemental Information, Fig. S6b–d). The observed rearrangements within the syntenic scaffolds included mainly deletions. Transposons were found on scaffolds carrying *PKS*- and *NRPS*-coding genes, suggesting that these genes can be influenced by transposable elements. 47% (52/110) of *PKS-* and 34% (14/41) *NRPS*-containing scaffolds possessed LTR signatures (Supplemental Information, Tables S6 and S7). Taken together, these results indicate that *PKS* genes have diversified in each Symbiodiniaceae species by several evolutionary processes.

### Phylogenetic analysis of adenylation and condensation domain subtypes (^L^C_L_, ^D^C_L_, Cyc and dual) in NRPS proteins

**Figure 3:**
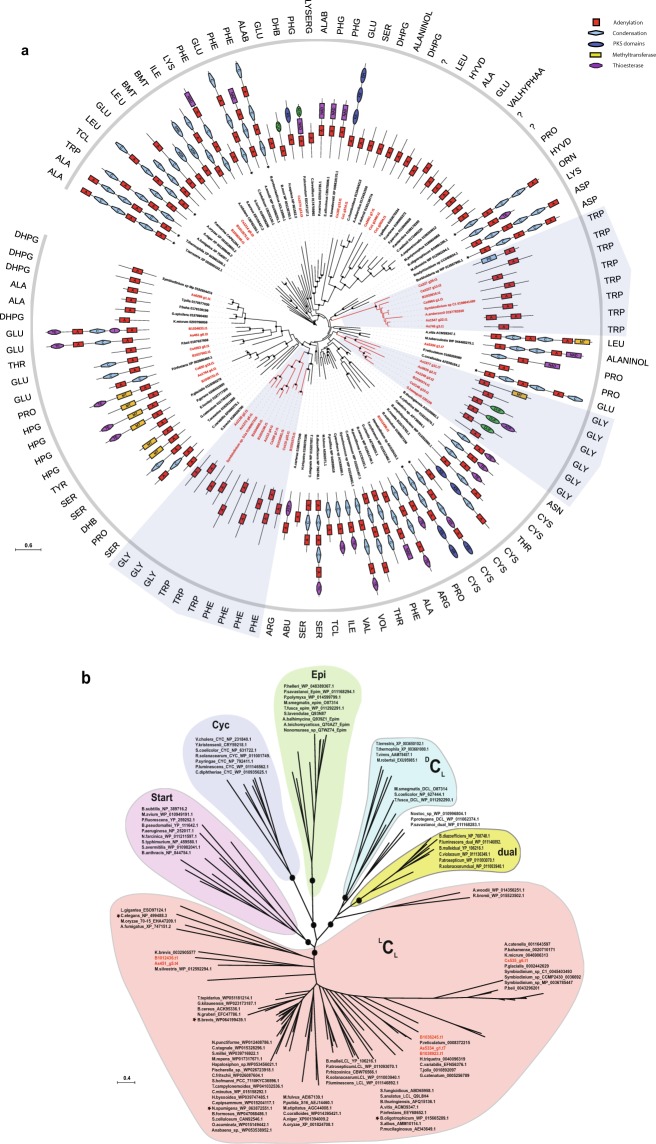
Phylogenetic analysis of adenylation (A) and condensation (C) domains of prokaryotic and eukaryotic NRPS. Dots indicate posterior probability ≥0.70 generated by Bayesian inference. (**a**) Analysis of adenylation domains shows specificity of monofunctional domains from the family Symbiodiniaceae toward three amino acids (glycine, tryptophan, and phenylalanine) as indicated by shaded regions. The specificity of the A-domain was determined using the Latent Semantic Indexing of the LSI-based A-domain predictor^28^. Colored blocked display domain organization and asterisks indicate multifunctional proteins that are too long to display. Details of protein sequences are provided in Supplemental Information, Table S4. (**b**) Condensation domains from Symbiodiniaceae belong to the ^L^C_L_ type (shown in red). Asterisks indicate sequences with different specificities beside group subtype specificity. (Epi = epimerization domain, dual = dual/epimerization domain, Cyc = cyclization domain).

To understand if freestanding A-domains identified in Symbiodiniaceae genomes follow the same non-ribosomal code of traditional NRPS systems^27^, we performed a phylogenetic comparison involving 117 adenylation sequences from different taxa. In addition, the amino acid substrate of adenylation domain was predicted by latent semantic indexing method^28^. One major observation was that freestanding A-domains appear in three major nodes (BI posterior probability: 1.00 & maximum likelihood probability: 72–100) that utilize tryptophan, glycine, and phenylalanine as substrates (three highlighted groups in Fig. 3a, Supplemental Information, Figs S7 and S8). In contrast, other proteins with di- or multi-domains displayed affinity for various substrates (Fig. 3a).

Phylogenetic analysis of condensation domains was dominated by functional categories of C-domains rather than species phylogeny or substrate specificity alone (Fig. 3b). Classification of C-domains by NaPDoS software indicated that Symbiodiniaceae genomes are rich in ^L^C_L_ subtypes (BI posterior probability: 0.73), that catalyze the condensation of two L-amino acids (Fig. 3b, Supplemental Information, Figs S9, S10), in contrast to a ^D^C_L_ that links an L-amino acid to a D-amino acid. Our survey revealed the presence of six condensation domains with the consensus motif (HHxxxDG) being maintained, except for G being substituted with L and N in B1036245.t1 and Cs535_g6.t1, respectively (Fig. 3b). The phylogeny also supports the close relationship between ^L^C_L_ and starter C-domains and dual and ^D^C_L_ domains, as previously reported in bacterial genomes, confirming the reliability of our analysis^29^. These results demonstrate the specificity of NRPS genes for specific amino acids, thus introducing a degree of chemical diversity in non-ribosomal peptide biosynthesis.

### Identification of metabolites and biosynthetic gene clusters from Symbiodiniaceae genomes

Polyols were identified based on their high-resolution mass data, as summarized in Beedessee *et al*. (ref.^16^). Doubly charged ions (negative ions) were searched for larger polyols (>2600 Da) in the MS spectra. Sample A3 showed the presence of zooxanthellatoxin-B (ZT-B), albeit in small amount, with an m/z of 1414.74 for the [M-2H]^2−^ (Supplemental Information, Fig. 11a). Only zooxanthellamide D (ZAD-D) could be identified from sample B1 with extracted ions at *m/z* 1050.57 for the [M + H]^+^ (Supplemental Information, Fig. 11b). No SCCs could be identified from sample C despite presence of many polyols (Supplemental Information, Figs 11c–12a). Samples B1 and C also showed similar LC-MS profiles and contained some identical unknown SCCs in the molecular weight range of 2,600–2,850 Da (Supplemental Information, Fig. 12b). It should be noted that other polyhydroxy SCCs were also detected in the crude methanol extracts of all samples and none of them corresponds to known zooxanthella polyhydroxy molecules^16^.

**Figure 4:**
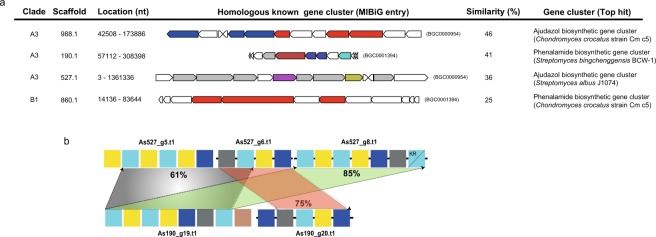
Multifunctional *PKS* genes in Symbiodiniaceae. (**a**) Table showing homologous gene clusters and similarities of different scaffolds from three clades obtained using antiSMASH version 4.1.0. Details of each gene cluster can be obtained using the MIBiG (Minimum Information about a Biosynthetic Gene cluster) entry number and is accessible at https://mibig.secondarymetabolites.org/repository.html. (**b**) Homology comparison of two scaffolds (527.1 and 190.1 of clade A3) shows an example of module duplication. Numbers indicate the percentage of identity shared between sequences. Details of modules are depicted in Fig. 1.

Analysis using antiSMASH on the three Symbiodiniaceae matched four PKS/NRPS-containing clusters to known biosynthetic gene clusters, with similarities between 25–46% (Fig. 4a). Clade A3 harbors a gene cluster with similarity to ajudazol and phenalamide biosynthetic genes from *Streptomyces* species and *Chondromyces crocatus* while clade B1 shares similarity with a phenalamide biosynthetic cluster from *Chondromyces crocatus*. High sequence similarity was noted in clade A3, offering an example of module duplication between modules of gene models in one scaffold, as well as between modules of different scaffolds (Fig. 4b). To examine the localization of KS protein, antibodies against the KS domain were used. Immunolocalization showed that KS proteins were mainly associated with reticulate chloroplasts in clade C (Supplemental Information, Fig. 13), although the possibility remains that KS proteins are localized in other organelles. Similar observations on the location of KS proteins in chloroplasts have been reported in *Karenia brevis*^30^.

## Discussion

### Evolution of modularity within Symbiodiniaceae genomes

**Figure 5:**
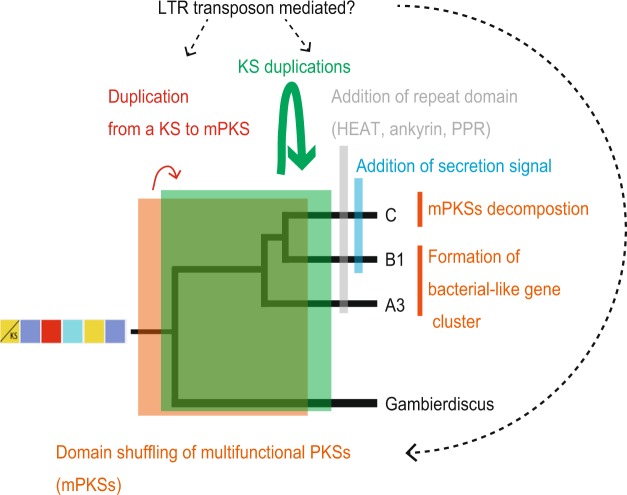
Evolution of *KS* gene in dinoflagellates. Several mechanisms may have contributed to biosynthetic diversification in Symbiodiniaceae. Bacterial-like gene clusters can be conserved and retained in several species. Decomposition of such multifunctional polyketide synthases and extensive duplication may have been mediated by LTR transposons, resulting in addition of secretory signals and repeat domains in the three clades (A3, B1 and C). On the other hand, duplication of a hybrid PKS-NRPS was not detected.

The genomic analysis presented here reveals expanded genetic diversity of metabolite-producing capacity in Symbiodiniaceae dinoflagellates. The polyketide biosynthesis machinery gains its functional and genetic modularity by changes through combinatorial events assisted by gene duplication, horizontal gene transfer (HGT), and recombination^31^. Our analysis shows that domain as well as module duplications established an important evolutionary mechanism toward modularity (Fig. 4b). Dinoflagellate genomes are scattered with large numbers of repeats, with frequent recombination events, and possess genes with high copy numbers due to duplication^17–19^. These features might have led to decomposition of Type I multifunctional PKS clusters, a phenomenon involving shuffling of domains and modules previously observed in bacteria^7^. However, there is increasing evidence of multifunctional PKS domains in several dinoflagellates, indicating that multifunctionality coevolves with monofunctional domains^16,23,32^. Our data show that monofunctional PKSs are related to multifunctional PKS (Fig. 1) but it remains unclear whether fusion of monofunctional PKS domains led to multifunctionality or *vice versa*. Retrotransposons may have been important contributors in the expansion of PKS and NRPS, since 34% and 47% of the scaffolds, respectively, are predicted to contain LTR signatures (Fig. 5, Supplemental Information, Table S6). Retrogenes account for >20% of all genes in Symbiodiniaceae clades^33^. The Ty1/copia LTR retrotransposon has been proposed as a likely candidate driver for retroposition in *Oxyrrhis marina*^34^.

HGT has been suggested as a significant event contributing to gene innovation with recent evidence linking HGT to various biological processes^35^. HGT is thought to contribute to genome innovation in *Fugacium kawagutii*, with 41 out of 56 potential HGT genes being of marine bacterial origin^18^. Gene transfer of *PKS* genes has been suggested in *Karenia brevis*^36^. Multiple rounds of intra- and intergenic gene duplication have been associated with the expansion of the light-harvesting complex (LHC) gene family in *Breviolum minutum* B1, suggesting gene conversion and/or genome rearrangement as an impetus for diversification^37^. Interestingly, monofunctional, probably *trans*-acting domains of either PKS or NRPS, are often fused with repeats units like HEAT (*h*untingtin, *e*longation factor 3, *A* subunit of protein phosphatase 2A and *T*OR1), ankyrin and pentatricopeptide (PPR) repeats (Fig. 5). HEAT repeats have been found in transport-related proteins while the ankyrin repeat family is the second largest dinoflagellate protein family in *Breviolum minutum* and is known to facilitate protein-protein interactions involved in several intracellular biological processes^17,38–40^. On the other hand, PPR proteins are nuclear-encoded, but are targeted to plastids and mitochondria, where they are involved in RNA processing and editing^41–43^.

### Evolution of polyketide biosynthesis

Fatty acid synthesis is probably carried out by type II FAS in dinoflagellates, based on a clear distinction between genes involved in fatty acid and polyketide biosynthesis^24^. Our data show that PKS domains have undergone extensive diversification in all three Symbiodiniaceae genomes. A plausible explanation for this expansion might be their involvement in novel functions, supported by the fact that ~15% of KS and ~9% of AT proteins possess targeting signal peptide, directed towards different organelles (Fig. 5). An FAS-like multi-domain polyketide synthase has been identified in *Durinskia baltica*^44^, associated with fatty acid biosynthesis. A recent transcriptomic survey of the dinoflagellate *Hematodinium* sp. revealed only Type I FAS^45^, while another study on *Gambierdiscus* spp. revealed a distinct Type II FAS system along with single KS domains^23^, suggesting a uniqueness of these pathways to specific dinoflagellates. Both Type I and Type II FAS systems can exist, as in *Toxoplasma*^46^. Some taxa possess only cytosolic type I, as in *Cryptosporidinium parvum*, while others have only plastid Type II, as in *Plasmodium falciparum*^47^. Clearly, apicomplexan and dinoflagellate ancestors possessed both systems.

AT domains of *cis*-AT display specificity towards various extender units (e.g. methylmalonyl-CoA, hydroxymalonyl-ACP, methoxymalonyl-ACP, etc) while *trans*-ATs are specific for malonyl-CoA. Stand-alone AT proteins have been reported in several PKSs with modules lacking AT domains and these proteins provide malonyl building blocks for the ACP domains of PKS^48,49^. Our analysis shows that these stand-alone *trans*-AT proteins are dominant in Symbiodiniaceae genomes, forming a major group that may undergo independent evolution compared to canonical *cis*-AT domains. The existence of such *cis-* and *trans*-AT has been reported in bacteria and interpreted as proof of independent evolution^50^. Bacterial *cis-*AT PKS have evolved mainly via module duplication and horizontal/vertical acquisition of entire assembly lines^7^, while *trans*-AT tends to recombine and to form novel gene clusters in a mosaic-like fashion^51^, as seen in the global pattern of AT in Symbiodiniaceae genomes (Fig. 2). Shelest *et al*. (ref.^8^) found that noniterative PKSs in algae depend mainly on *trans*-AT and are features of multimodular PKS. Interestingly, we observe fragments of genes have been retained even between dinoflagellate genera (e.g. Dinoflagellate PKS-III in Fig. 1), attesting how several evolutionary events such as gene duplication and domain shuffling, with help of repeat domains and LTR retrotransposition have promoted diversification of *PKS* genes (Fig. 5).

### Evolution of non-ribosomal peptide biosynthesis

Few studies have reported NRPS in dinoflagellate transcriptomes^52,53^; however, detailed analyses of NRPS remain limited. To our knowledge, this is the first study that look at the role and affinities of adenylation and condensation domains in dinoflagellates. Compared to Type I PKS, NRPSs were reduced in number. NRPSs are known to be less abundant in eukaryotic microalgae^8^. A sequence of amino acids within the A-domain catalytic pocket appears to govern recognition and activation of an amino acid substrate. Thus, any point mutations within this segment can drastically change the specificity of the A-domain. A mono-modular adenylation domain favors incorporation of polar and non-polar amino acids during peptide synthesis (Fig. 3a). A conserved domain organization in mono/bi-modular NRPSs exists in fungal species, implying that this architecture is critical for its function^54^. Single A- or A-T domains can interact with other NRPS components to achieve biosynthesis by successful activation and transfer of the substrate to the C domain in either the same or different NRPS^55^. NRPSs are mainly modular enzymes with several domains; however, there are reports of nonmodular enzymes among fungal subfamilies^54,56^.

### Conserved secondary metabolic pathways in the family Symbiodiniaceae

Symbiodiniaceae lineages diversified from the ancestral clade A ~160 MYA, at the beginning of the Eocene^1,57^ and have adapted to different environments, performing critical functions in reef ecosystems, as well as serving as photosynthetic endosymbionts of different phyla^15^. New genomes now allow us to compare biosynthetic pathways, shedding light on the organization and role of pathways and their contribution to ecological success. Several biosynthetic gene clusters are conserved between *Symbiodinium tridacnidorum* (clade A3), *Breviolum minutum* (clade B1), and *Cladocopium sp*. (clade C) (Supplemental Information, Fig. S6b–d), despite the divergence time^1^. Rosic *et al*. (ref.^58^) reported the importance of conserved phosphatidylinositol signaling pathways in four Symbiodiniaceae clades and their contribution to symbiotic interactions. We found that clades A3 and B1 produce unique polyketides, supporting the clade-metabolite hypothesis^13^. Metabolite profiles of different Symbiodiniaceae species are influenced by different temperatures and light regimes^59^. On the other hand, metabolomic similarity was detected only between clades B1 and C. At this stage, it is difficult to link specific metabolites to specific pathways, but these results suggest that novel pathways must have evolved in the common ancestor of clades B1 and C to provide a common set of metabolites, irrespective of their hosts and environments. Biological systems regulate biochemical and cellular processes when subjected to environmental changes^60^. This study shows that Symbiodiniaceae genomes encode PKS and NRPS enzymes with broad substrate tolerance as a cost-effective way of generating chemical diversity.

The Screening hypothesis suggest that organisms that produce many chemicals, have more chances of enhanced fitness because greater chemical diversity increases the chance of producing metabolites with unique traits, as illustrated by zooxanthellatoxins and zooxanthellamides^61^. So why are only a few major pathways conserved among these species? It might be beneficial for organisms to elongate existing pathways to generate new chemical diversity, instead of originating entirely new pathways^62^. Dinoflagellates are known to form harmful algal blooms, that negatively affect ecosystems via the accumulation of toxins through food webs that can cause classical seafood poisoning. Thus, insights into their biosynthesis can provide new ways for detection of toxin in environmental samples^63^. From a biotechnological perspective, such novel polyketide biochemistries can provide valuable tools for the combinatorial biosynthesis of future medicines^64^.

In conclusion, we surveyed three genomes for genes associated with secondary metabolism. We showed that PKS genes are more diversified than NRPS genes and that several evolutionary processes have contributed to this diversification. Furthermore, these genes displayed a degree of substrate specificity and flexibility that has been maintained evolutionarily, irrespective of host system. These results demonstrate that Symbiodiniaceae genomes are well equipped to generate chemical diversity when it comes to secondary metabolite biosynthesis. This comparative genomic study provides preliminary insights into how dinoflagellate genomes adapt to hosts’ environment and addresses the functional roles of secondary metabolites in such symbiotic relationships.

## Methods

### Symbiodiniaceae cultures

*Breviolum minutum* (Clade B1, strain Mf1.05b) was isolated from the stony coral, *Montastraea (Orbicella) faveolata* by Dr. Mary Alice Coffroth (University of New York, Buffalo, USA) and *Symbiodinium tridacnidorum* (clade A3, strain Y106) and *Cladocopium* sp. (clade C, strain Y103) were isolated from the clam *Tridacna crocea* and bivalve *Fragum* sp., respectively, by late Dr. Terufumi Yamasu (University of the Ryukyus, Okinawa, Japan). Cultures were maintained in autoclaved, artificial seawater containing 1X Guillard’s (F/2) marine-water enrichment solution (Sigma-Aldrich: G0154), supplemented with antibiotics (ampicillin (100 μg/mL), kanamycin (50 μg/mL), and streptomycin (50 μg/mL)). Culturing and sampling were performed according to the protocol of Shoguchi *et al*. (ref.^17^).

### Data retrieval

Throughout this manuscript, we adopted the revised terminology^1^ but retain the previous familiar clade terminology and tag gene models from the three Symbiodiniaceae genomes (A3, B1 and C) with the letters A, B, and C to improve the readability and interpretation. To understand diversification and molecular evolution of PKS and FAS, we performed an extensive search for *PKS* (*KS* & *AT*) and *FAS* (*FabB-KASI*, *FabF-KASII* & *FabD*) genes within three Symbiodiniaceae genomes, as these domains are conserved^65^. The genome browser MarinegenomicsDB (http://marinegenomics.oist.jp/genomes/gallery/) and the *Fugacium kawagutii* browser (http://web.malab.cn/symka_new/genome.jsp) were accessed in order to retrieve PKS (KS & AT), FAS (FabB-KASI, FabF-KASII & FabD) and NRPS (A & C) sequences from clades A3, B1, and C and *Fugacium kawagutii*, respectively^18,66^. In addition, transcriptome datasets for several dinoflagellates, apicomplexans, stramenopiles, and haptophytes were downloaded from the Marine Microbial Eukaryote Transcriptome Sequencing Project (MMETSP) (http://datacommons.cyverse.org/browse/iplant/home/shared/imicrobe/camera) and surveyed for comparative analysis^67^. Amino acid sequences of several other prokaryotes, fungal, animal, and chlorophyte PKS and NRPS domains were obtained from NCBI Genbank with additional sequences from dinoflagellates^23,68^. Further NRPS sequences from *Proteobacteria*, *Firmicutes*, and *Cyanobacteria* were obtained from Wang *et al*. (ref.^5^). Functional prediction and conserved active site residues in sequences were identified using Pfam^69^. Only PKS, FAS, and NRPS sequences with full domains and conserved active sites were used in the analysis. Details of gene IDs and their transcriptome support are provided in Supplemental Information, Tables S1–S4.

### Phylogenetic analysis

Type I and II PKS/FAS and condensation (C) & adenylation (A) domain sequences representing different taxa were used for Bayesian inference and maximum likelihood analysis. Four amino acid (aa) domain sequence datasets comprising of 233 KS sequences (226 aa), 96 AT sequences (208 aa), 117 A-sequences (400 aa), and 110 C-sequences (260 aa) were aligned using the MUSCLE algorithm^70^. Sites within alignments where homology was ambiguous (e.g. large insertions and deletions) were removed prior to phylogenetic analyses. Maximum likelihood phylogenetic analysis was performed using RaxML with 1000 bootstraps using the GAMMA and Le-Gasquel amino acid replacement matrix^71^. Bayesian inference was conducted with MrBayes v.3.2^72^ using the same replacement model and run to maximum of six million generations and four chains or until the posterior probability approached 0.01. Statistics and trees were summarized using a burn-in of 25% of the data. Using two methods provided a convenient way to verify different phylogenetic estimates, since each method has its intrinsic strengths and assumptions about the evolutionary process. Trees were edited using Figtree (http://tree.bio.ed.ac.uk/software/figtree/).

### *In silico* analysis of PKS and NRPS genes and genomic locations

Monomer prediction based on specificity of the A-domain was determined using the Latent Semantic Indexing of the LSI-based A-domain predictor^28^. NaPDos was used to determine C-domain types^73^. For AT domains, sequences were compared to the Hidden Markov Model-based ensemble (HMM) generated by Khayatt *et al*. (ref.^25^). Additional information about substrate specificity was detected using I-TASSER^74^. AntiSMASH (Antibiotics & Secondary Metabolite Analysis SHell) version 4.1.0 was used with default settings to identify *NRPS* and *PKS* gene clusters within scaffold regions using nucleotides sequences as queries^75^. Subcellular localization of PKS gene products toward organelles (e.g. chloroplast and mitochondria) or the presence of signal peptide or membrane anchor was determined using ChloroP 1.1 and TargetP 1.1 using a cut-off score of ≥0.50 each and the subcellular localization predictor, DeepLoc^76–78^. NUCmer operation of SyMap v4.2 (Synteny Mapping and Analysis Program) was used to align and visualize syntenic relationships between the three clades^79^. Scaffold information and descriptions of these genomes were imported into SyMap as GFFs (General Feature Files). To determine orthologs, we performed an all-against-all BLAST search of *PKS*-coding scaffolds of one genome against itself at a BLAST bit score cutoff of ≥100 and e-value ≤ e^−20^. Outputs were parsed and processed, and orthologous pair detection was conducted using custom perl scripts. Possible segmental duplications were visualized using Circos^80^. GC content variations in *PKS*-coding scaffolds were analyzed using GC-profile with a halting parameter of 100^81^. LTR Finder 1.05 was used with defaults parameters to search for long terminal repeat (LTR) retrotransposon-specific features^82^.

### Polyol extraction from Symbiodiniaceae cultures

All cultured biomass samples were treated as previously described^8^. Cultured cells were collected by centrifugation (9,000 × g and 14,000 g, 10 min, 10 °C). After discarding the supernatant, a cell pellet was extracted with methanol (three times) at room temperature. Methanol (400 μL) was added to the biomass followed by vortexing (1 min), sonication (10 min), and centrifugation (14,000 × g, 10 min, 10 °C) to give a methanol extract. The resulting clear solution was transferred into a new tube. By adding methanol (400 μL) to the residue, a 2^nd^ methanol extraction was conducted in the same fashion. The 2^nd^ clear methanol extract was again collected and stored at −30 °C. Additional methanol (400 μL) was added to the residue, vortexed (1 min), and kept overnight at room temperature. After centrifugation, the 3^rd^ methanol extract was pooled with the previous extracts (total 1,200 μL), and marked as crude extract. To remove lipophilic materials from the crude extract, an aliquot (50 μL) of the crude extract was suspended in 50 μL water-methanol (90:10) containing 0.5% formic acid. The suspension was vortexed (30 sec) and centrifuged (14,000 × g, 10 min, 10 °C) to give a clean solution. The clean solution was transferred into a new tube (the stock solution) and the insoluble part was discarded. The stock solution was kept at −30 °C before NanoLC-MS analysis or immediately analyzed after dilution. All crude extracts were lyophilized and stored at −30 °C.

### NanoLC-MS analysis of Symbiodiniaceae methanol extract

A Thermo Scientific hybrid (LTQ Orbitrap) mass spectrometer was used for MS data collection. The mass spectrometer was equipped with a HPLC (Paradigm MS4, Michrom Bioresources Inc.), an auto-sampler (HTC PAL, CTC Analytics), and a nanoelectrospray ion source (NSI). The high-resolution MS spectrum was acquired at 60,000 resolution in FTMS mode (Orbitrap), full mass range *m/z* 400–2,000 Da with capillary temperature (200 °C), spray voltage (1.9 kV), and both positive and negative ion modes were used. The lipid-depleted crude extract (stock solution) was diluted (1:50) by adding water-methanol (50:50) containing 0.25% formic acid and separated on a capillary ODS column (50 × 0.18 mm, 3 μm, C_18_, Supelco). A 20-min gradient (10% B for 0–2 min, 10–100% B for 2–10 min, hold 100% B for 10–15 min, equilibration 10% B for 15.1–20.0 min, where solvent A was water: acetonitrile 98:2 and solvent B was water: acetonitrile 2:98, both containing 0.1% formic acid; flow rate 2.0 μL/min, injection, 2.0 μL) was used for polyol separation.

### KS protein localization

KS protein localizations were visualized using a modified version of the protocol of Berdieva *et al*. (ref.^83^). Briefly, cells were prefixed in methanol: F/2 medium (1:1) at RT for 15 min. Samples were then fixed in methanol at −20 °C overnight. Cells were washed in PBS, followed by permeabilization with 1% Triton X-100 for 15 min (5 min for clade B1), further washed with PBS and blocked with 5% normal goat serum-PBST for 1 h. Subsequently cells were incubated overnight at 4 °C with primary anti-KS antibodies at a 1:100 dilution in blocking solution. Primary antibody solution was then removed with 3 × 5-min PBS washes and cells were incubated with Alexa Fluor 488 (Abcam Cat #ab150077) secondary antibody for 1 h at RT (1:100 in blocking solution), ending with several PBS washes. Coverslips were mounted in DAPI-containing Vectashield on glass slides and visualized using a Zeiss Axio-Observer Z1 LSM780 confocal microscope under a Plan-APOCHROMAT 63X/1.4 oil DIC objective lens. Fluorescence excitation/emission wavelengths were 410/482 nm for DAPI, 499/614 nm for Alexa Fluor 488, and 649/740 nm for chlorophyll autofluorescence. Data were acquired using Zeiss ZEN version 14.0.8.201 software. For negative controls, primary antibodies were omitted. Z-stacks profiles were analyzed using ImageJ^84^. DIC imaging was performed using a Zeiss Image-Z1 under 40X.

## Supplementary information

Supplementary Information

## Data Availability

The datasets supporting the conclusions of this article are available are accessible in the DDBJ/EMBL/NCBI database with BioProject IDs PRJDB3242 (clade A3), PRJDB732 (clade B1), and PRJDB3243 (clade C), respectively. Raw data for metabolite profiling is accessible at the genome browser site (http://marinegenomics.oist.jp/gallery/).
