# Supplementary material for: Diversified secondary metabolite biosynthesis gene repertoire revealed in symbiotic dinoflagellates
Source: Sci Rep. 2019 Feb 4;9:1204. doi: 10.1038/s41598-018-37792-0 (PMC6361889; doi:10.1038/s41598-018-37792-0)
Supplement: Supplementary file 1 — Supplementary Information [file 41598_2018_37792_MOESM1_ESM.pdf]

Supplementary Information

Diversified secondary metabolite biosynthesis gene repertoire revealed in symbiotic dinoflagellates

Girish Beedessee[1*], Kanako Hisata[1], Michael C. Roy[2], Frances M. Van Dolah[3], Noriyuki Satoh[1], Eiichi Shoguchi[1*]

[1]Marine Genomics Unit, Okinawa Institute of Science and Technology Graduate University, Onna, Okinawa 904-0495, Japan.

[2]Instrumental Analysis Section, Okinawa Institute of Science and Technology Graduate University, Onna, Okinawa 904-0495, Japan

[3]College of Charleston, School of Sciences and Mathematics, 66 George St., Charleston, South Carolina, 29424, USA.

Supplementary Figures

Figure S1. Molecular phylogenetic tree of ketosynthase domain from prokaryotes and eukaryotes analyzed using Bayesian inference.

Figure S2. Molecular phylogenetic tree of ketosynthase domain from prokaryotes and eukaryotes analyzed using maximum likelihood.

Figure S3. GC plots of 4 scaffolds associated with dinoflagellate PKS-I (Fig. 1). Gaps less than 1% of the input scaffold fasta sequence were filtered. Plots generated using a halting parameter of 100. The GC profile is shown in red while segmentation points are depicted by the numbered green boxes.

Figure S4. Molecular phylogenetic tree of acyltransferase domain from prokaryotes and eukaryotes analyzed using Bayesian inference.

Figure S5. Molecular phylogenetic tree of acyltransferase domain from prokaryotes and eukaryotes analyzed using maximum likelihood.

Figure S6. Pathway duplication and conservation within and across *Symbiodinium* clades. **a** Plot showing duplicate gene distribution within *PKS*-containing scaffolds of three *Symbiodinium* genomes. Colored sections (black = clade B1, orange = clade C, blue = clade A3) represent scaffolds studied in Fig. 1. A link represents a possible duplication event between two domains. **b** Synteny plot of clade A3 and B1 *PKS*-containing scaffolds. **c** Synteny plot of clade B1 and C *PKS*-containing scaffolds. **d** Synteny plot of clade A3 and C *PKS*-containing scaffolds. Dotted boxes highlight regions of significant homology between genomes. Green colored dotted boxes show common regions shared among the three genomes.

Figure S7. Molecular phylogenetic tree of adenylation domain from prokaryotes and eukaryotes analyzed using Bayesian inference.

Figure S8. Molecular phylogenetic tree of adenylation domain from prokaryotes and eukaryotes

analyzed using maximum likelihood.

Figure S9. Molecular phylogenetic tree of condensation domain from prokaryotes and eukaryotes analyzed using Bayesian inference.

Figure S10. Molecular phylogenetic tree of condensation domain from prokaryotes and eukaryotes analyzed using maximum likelihood.

Figure S11. a. NanoLC-MS (negative ion) profile and mass spectrum (expanded) of the methanol extract of Clade A3. **b.** Nano-LC-MS (positive ion) profile and mass spectrum (expanded) of the methanol extract of Clade B1. **c.** NanoLC-MS (positive ion) profile and mass spectrum (expanded) of the methanol extract of Clade C.

Figure S12. a. NanoLC-MS (negative ion) profile and mass spectrum (expanded) of the methanol extract of Clade C. **b.** Similarity of nanoLC-MS (negative ion) profile and mass spectrum (expanded) of the methanol extract of Clade C and B1.

Figure S13. Immunofluorescent staining of *Symbiodinium* cells with anti-KS antibody. **a.** Differential interference contrast (DIC) imaging showing the spherical shape and internal complexity of cells at 40x magnification. **b-d.** Confocal images (63x) of clade C stained with KS antibody show localization of KS proteins (arrows). Nuclei are stained blue with DAPI (b), KS proteins are in green (c) and merged image of nuclei and KS protein staining (d) **e.** DIC imaging of cells at 63x showing detailed peripheral localization of chloroplasts (red autofluorescence) and nuclei (blue). **f-h.** Confocal images (63x) of control cells stained with only secondary antibody. Nuclei are stained with DAPI (f), no KS protein were stained (g) and merged image of nuclei and no-KS staining (h). White dotted lines show the cell outlines. Scale bars are 10 μm in the panels.

Supplementary Tables

Table S1: Ketosynthase domain containing genes in *Symbiodinium* clades used in this study.

Table S2: Acyltransferase domain containing genes in *Symbiodinium* clades used in this study.

Table S3: Condenstion domain containing genes in *Symbiodinium* clades used in this study.

Table S4: Adenylation domain containing genes in *Symbiodinium* clades used in this study.

Table S5: Prediction scores of ChloroP and TargetP, supported by DeepLoc subcellular localization predictor.

Table S6: Features of LTR-retrotransposons identified from PKS-associated scaffolds studied in this paper.

Table S7: Features of LTR-retrotransposons identified with NRPS-associated scaffolds studied in this paper.

Table S8: Details of NCBI, MMETSP and *Symbiodinium kawagutii* sequences belonging to the Dinoflagellate PKS clade (Green insert) in Fig. 1.

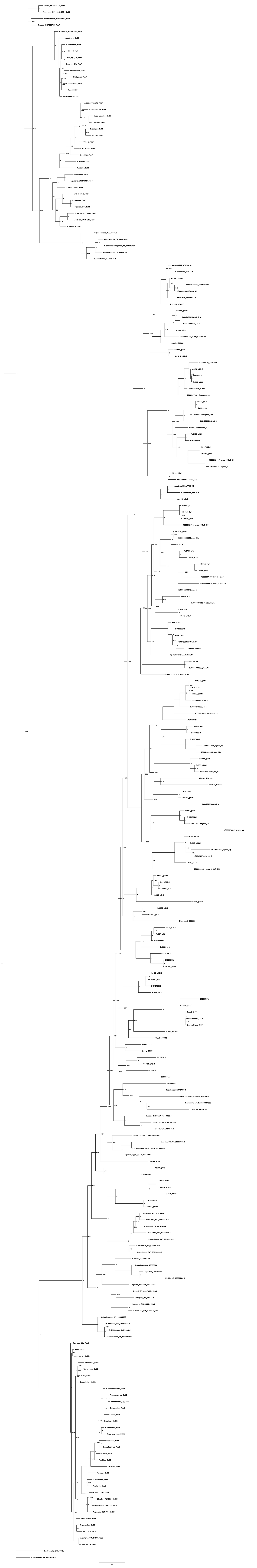

Figure S1: Molecular phylogenetic tree of ketosynthase domain from prokaryotes and eukaryotes analyzed using Bayesian inference. Details regarding Symbiodinium sequences are provided in Supplementary Table S1.

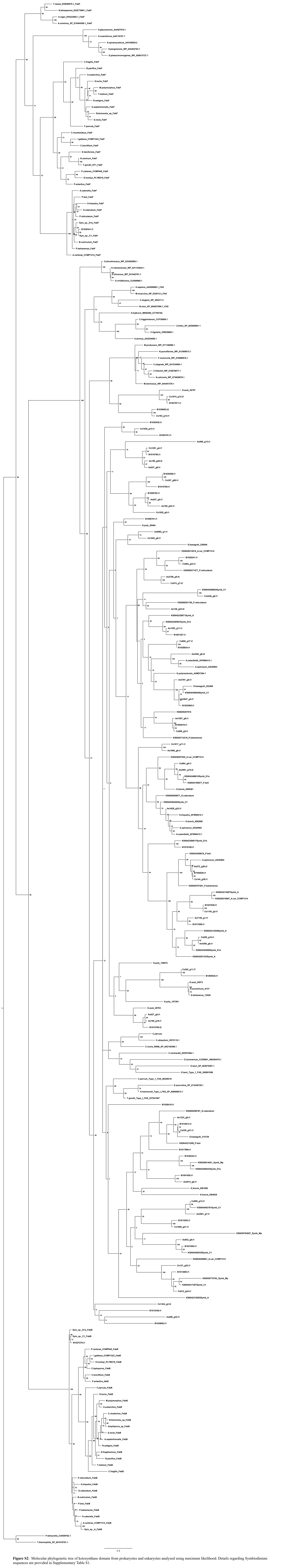

Figure S2: Molecular phylogenetic tree of ketosynthase domain from prokaryotes and eukaryotes analyzed using maximum likelihood. Details regarding Symbiodinium sequences are provided in Supplementary Table S1.

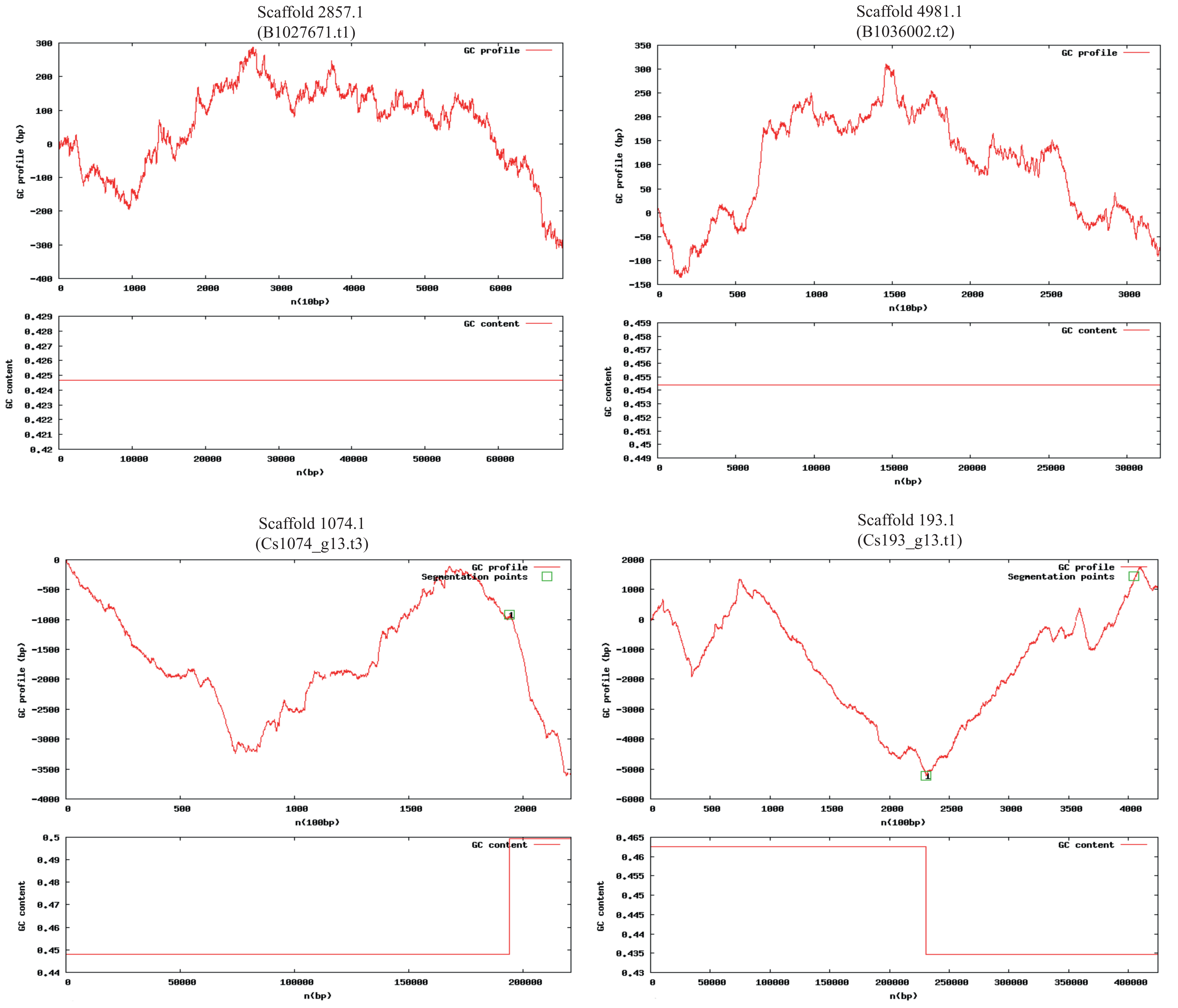

Figure S3: GC plots of 4 scaffolds associated with dinoflagellate clade PKS-I (Figure 1a). Gaps less than 1% of the input scaffold fasta sequence were filtered. Plots generated using a halting parameter of 100. The GC profile is shown by the red line while segmentation points are depicted by the numbered green boxes.

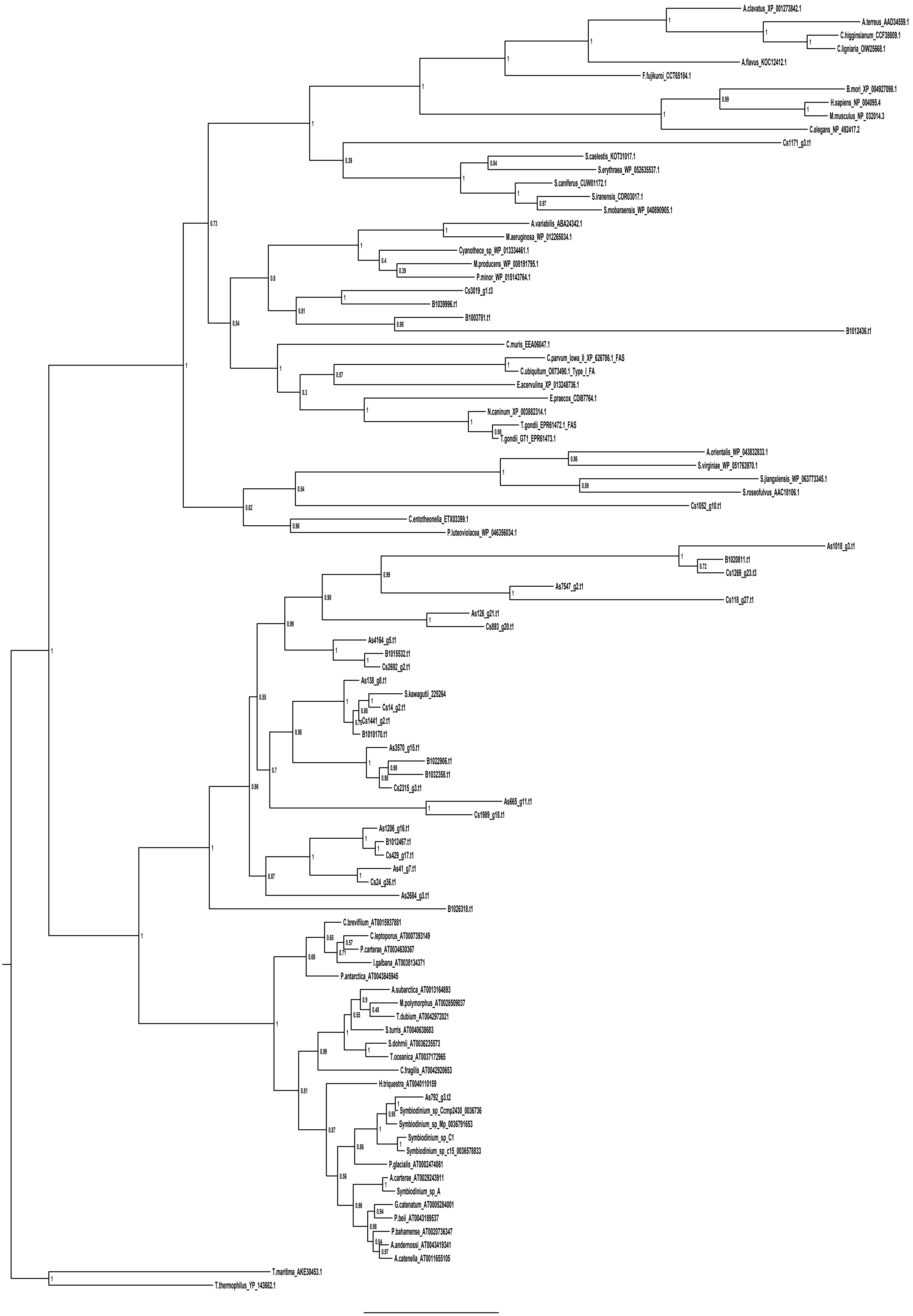

Figure S4: Molecular phylogenetic tree of acyltransferase domain from prokaryotes and eukaryotes analyzed using Bayesian inference. Details regarding Symbiodinium sequences are provided in Supplementary Table S2.

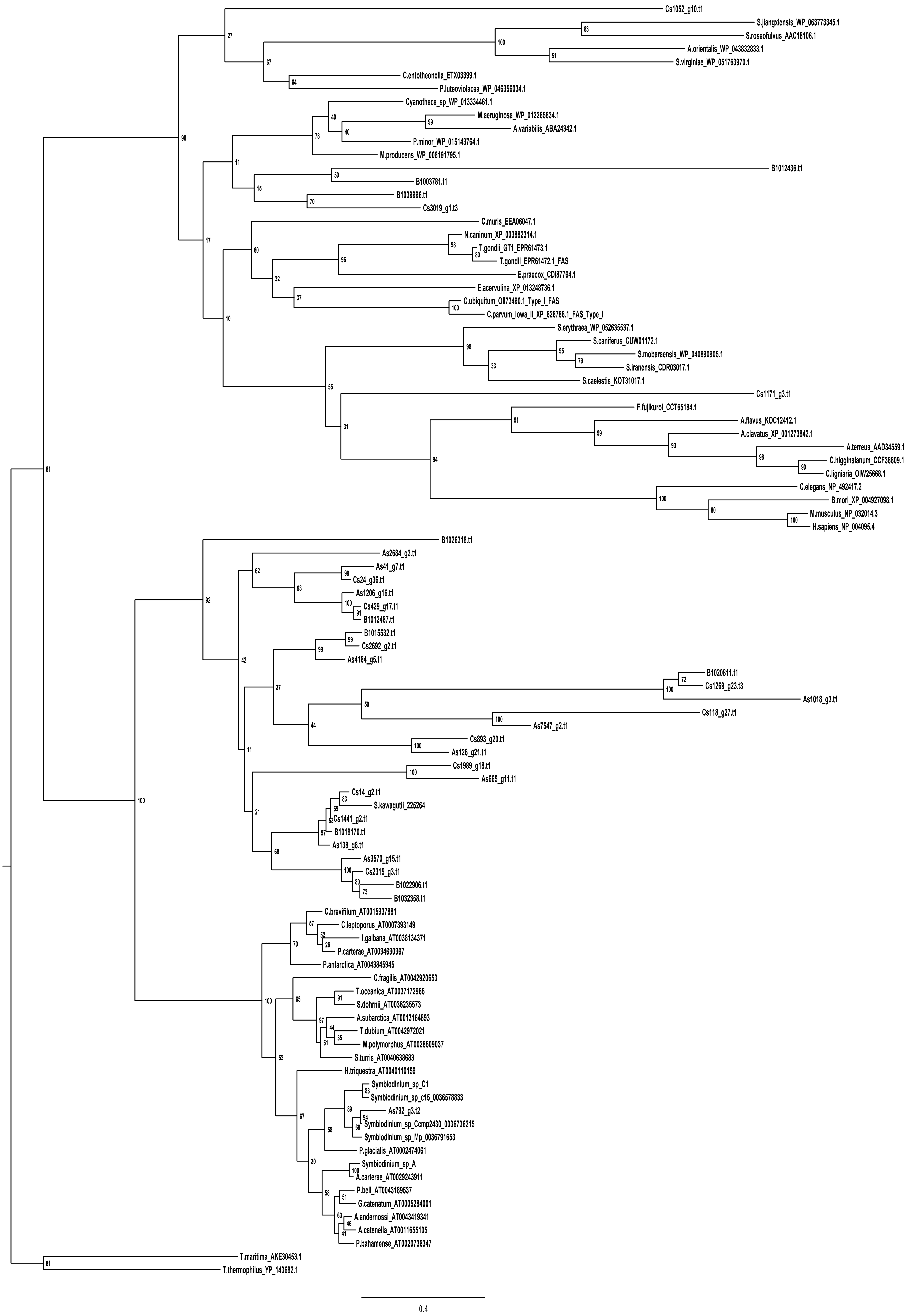

Figure S5: Molecular phylogenetic tree of acyltransferase domain from prokaryotes and eukaryotes analyzed using maximum likelihood. Details regarding Symbiodinium sequences are provided in Supplementary Table S2.

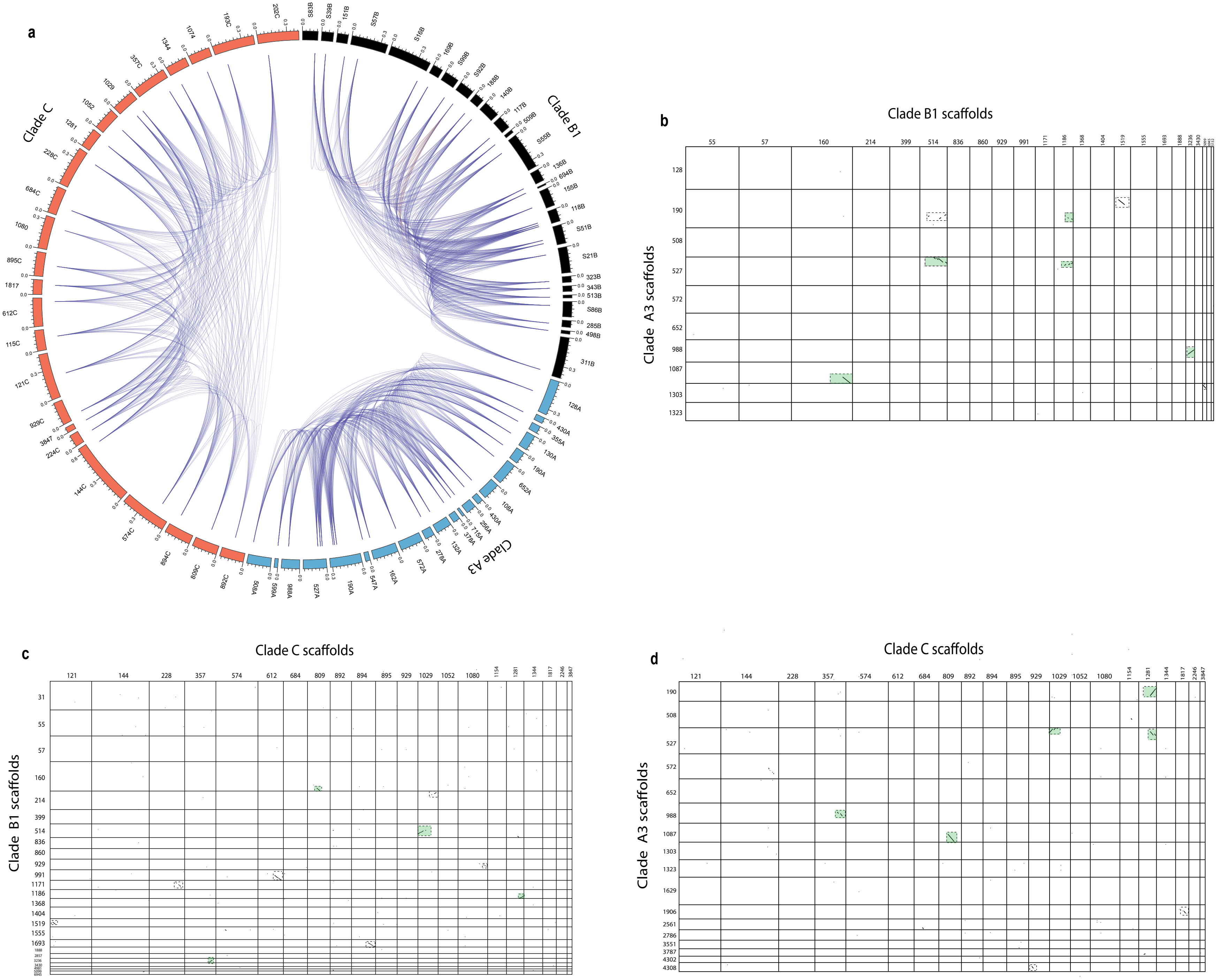

Figure S6. Pathway duplication and conservation within and across Symbiodinium clades. a Plot showing duplicate gene distribution within PKS-containing scaffolds of three Symbiodinium genomes. Colored sections (black = clade B1, orange = clade C, blue = clade A3) represent scaffolds studied in Fig. 1. A link represents a possible duplication event between two domains. b Synteny plot of clade A3 and B1 PKS-containing scaffolds. c Synteny plot of clade B1 and C PKS-containing scaffolds. d Synteny plot of clade A3 and C PKS-containing scaffolds. Dotted boxes highlight regions of significant homology between genomes. Green colored dotted boxes show common regions shared among the three genomes.

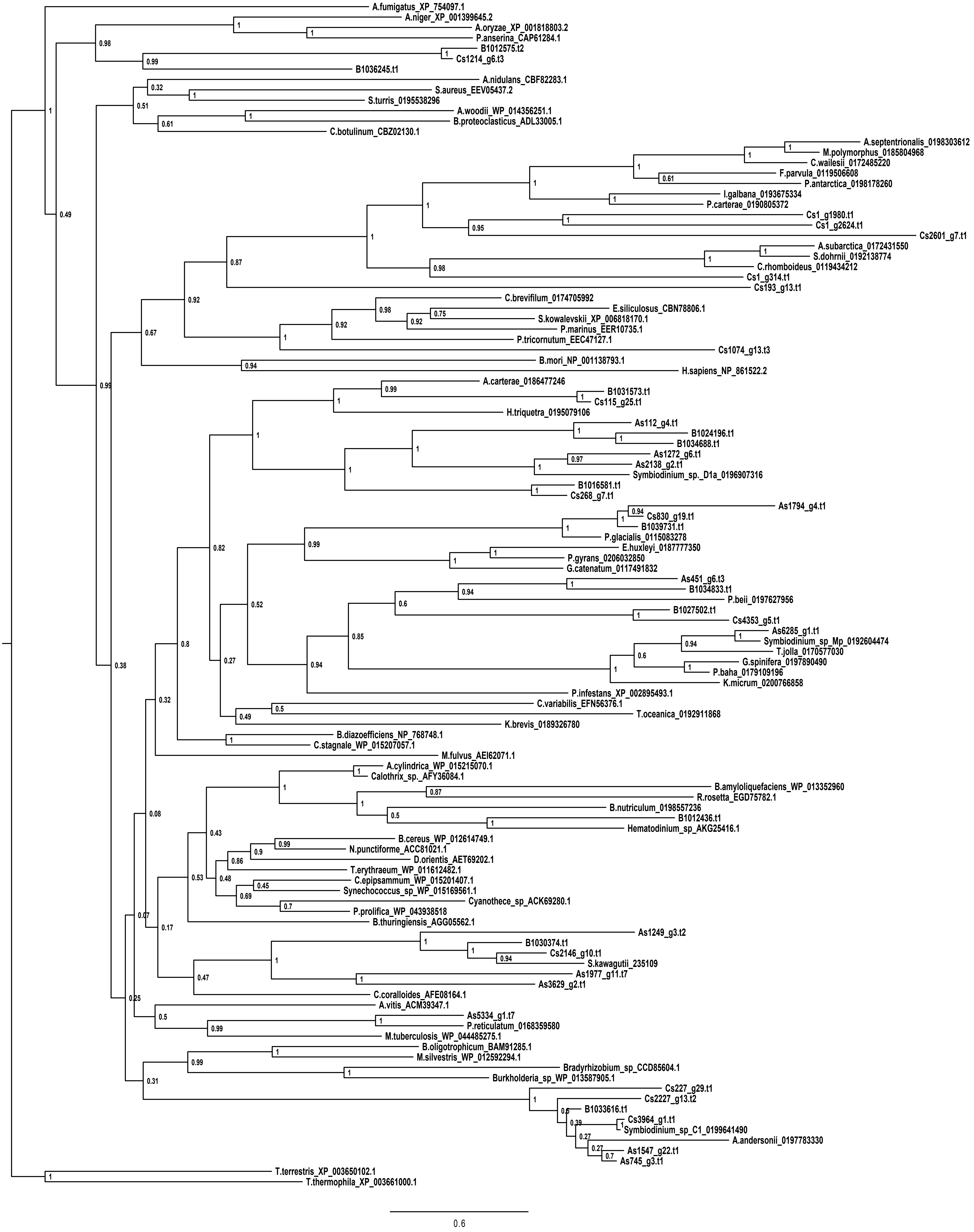

Figure S7: Molecular phylogenetic tree of adenylation domain from prokaryotes and eukaryotes analyzed using Bayesian inference. Details regarding Symbiodinium sequences are provided in Supplementary Table S4.

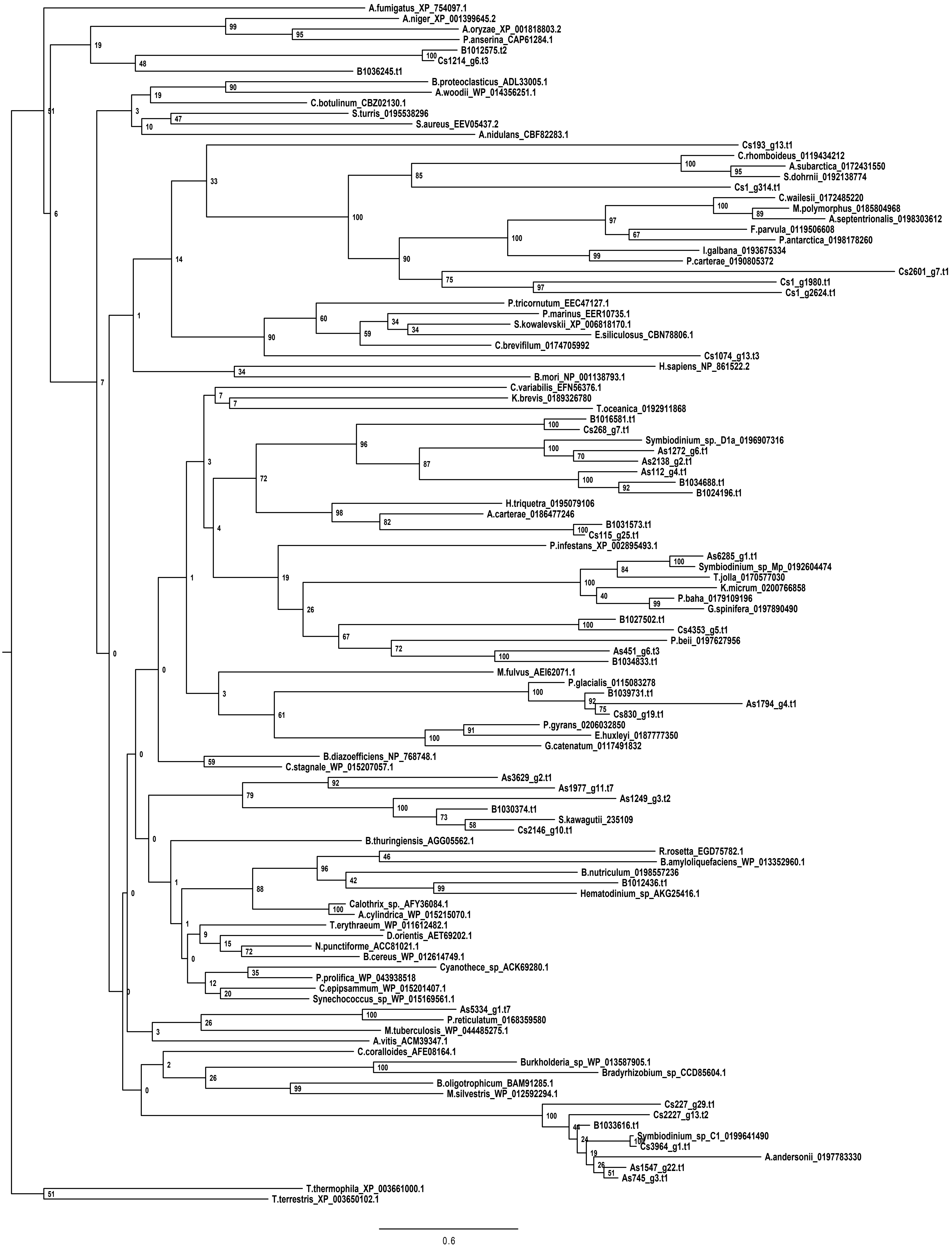

Figure S8: Molecular phylogenetic tree of adenylation domain from prokaryotes and eukaryotes analyzed using maximum likelihood. Details regarding Symbiodinium sequences are provided in Supplementary Table S4.

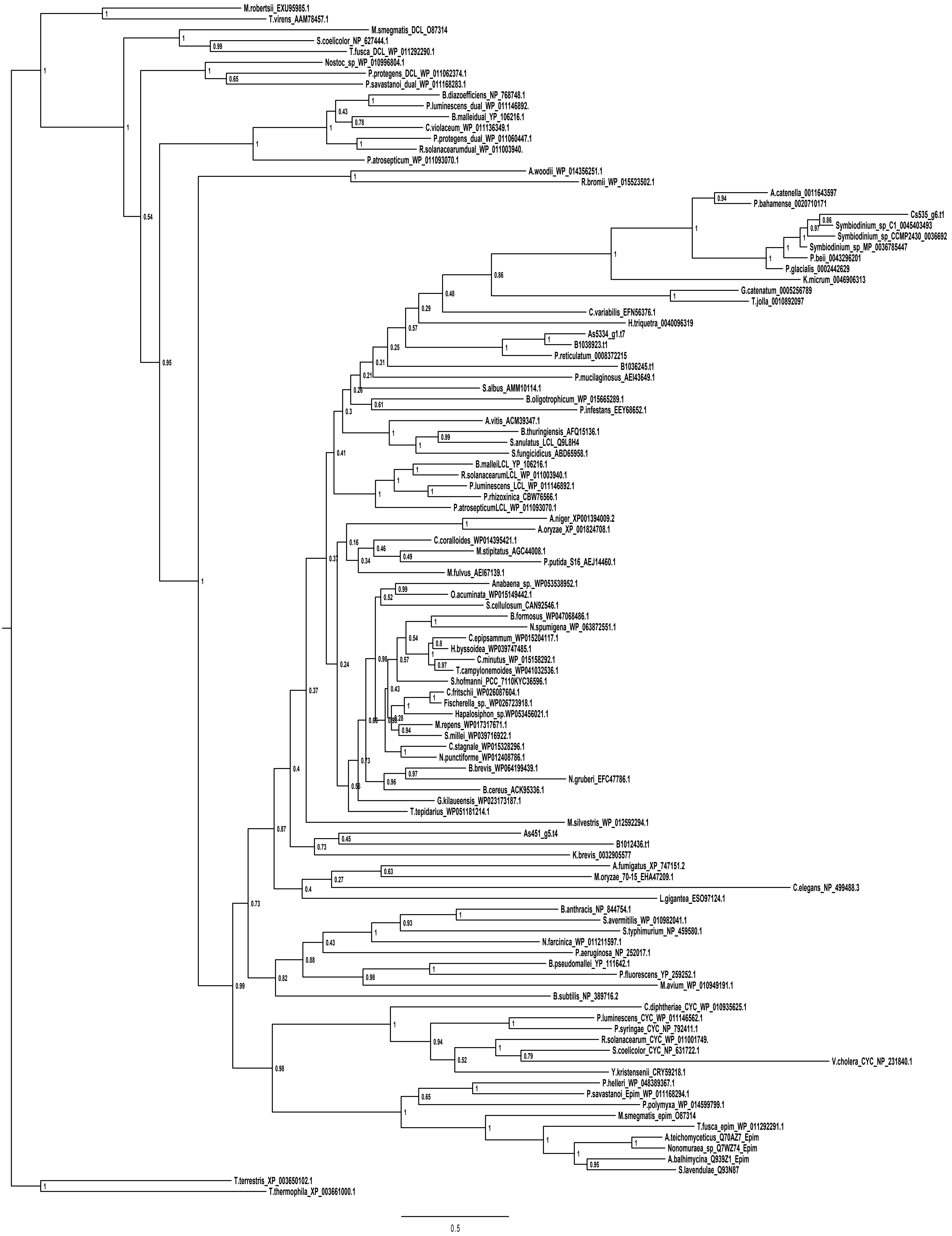

Figure S9: Molecular phylogenetic tree of condensation domain from prokaryotes and eukaryotes analyzed using Bayesian inference. Details regarding Symbiodinium sequences are provided in Supplementary Table S3.

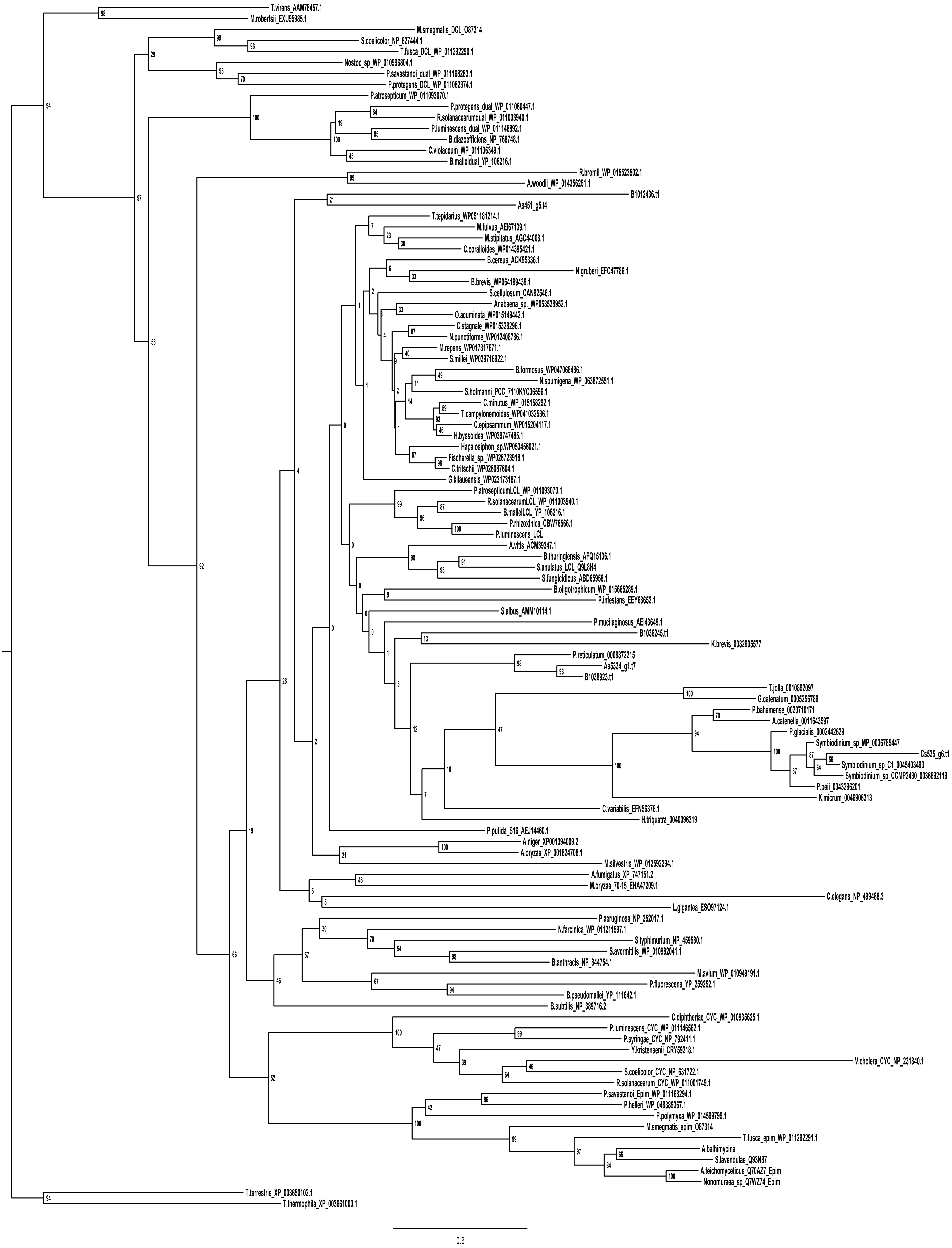

Figure S10: Molecular phylogenetic tree of condensation domain from prokaryotes and eukaryotes analyzed using maximum likelihood. Details regarding Symbiodinium sequences are provided in Supplementary Table S3.

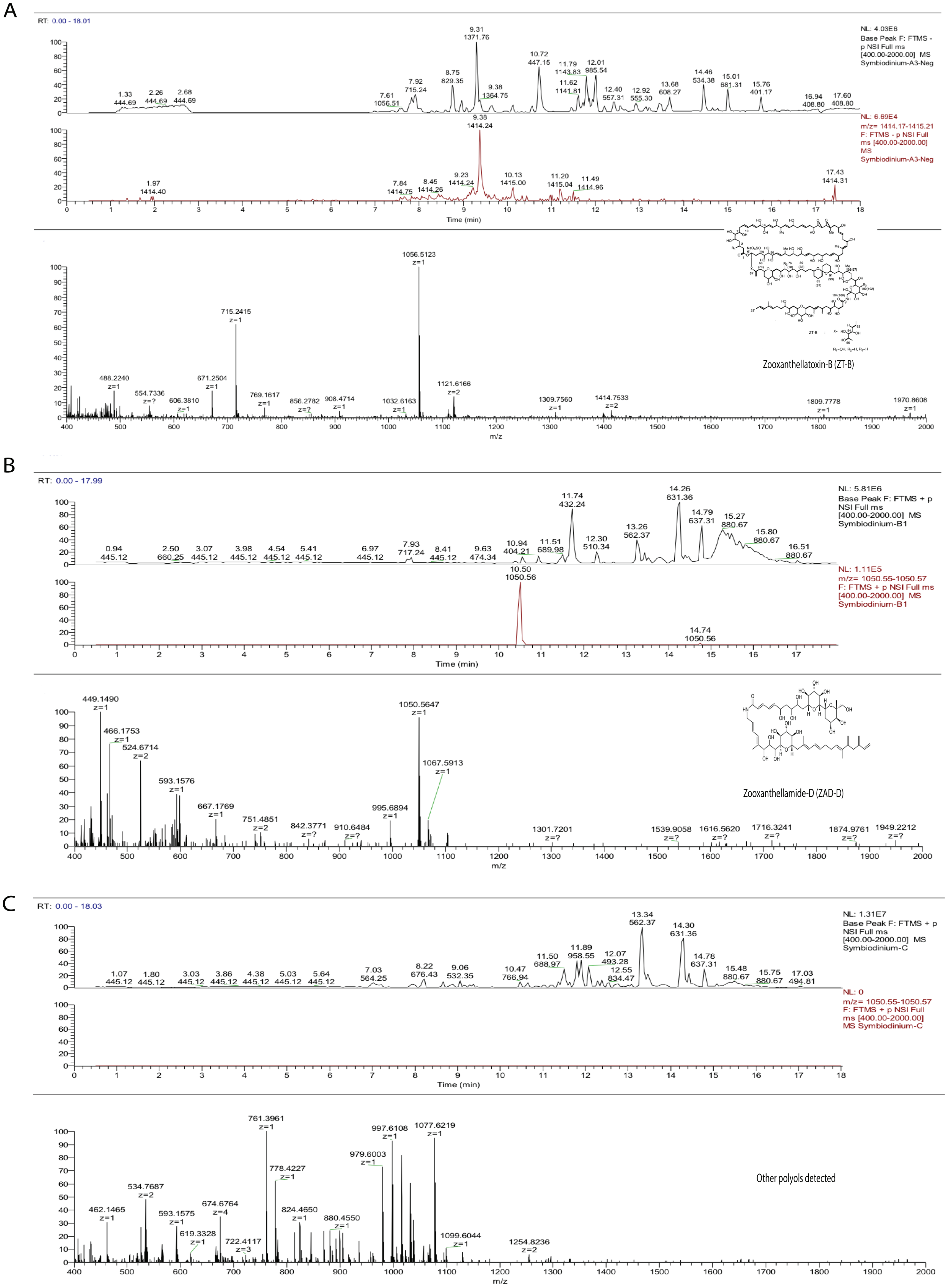

Figure S11: **a.** NanoLC-MS (negative ion) profile and mass spectrum (expanded) of the methanol extract of Clade A3. **b.** Nano-LC-MS (positive ion) profile and mass spectrum (expanded) of the methanol extract of Clade B1. **c.** NanoLC-MS (positive ion) profile and mass spectrum (expanded) of the methanol extract of Clade C.

a

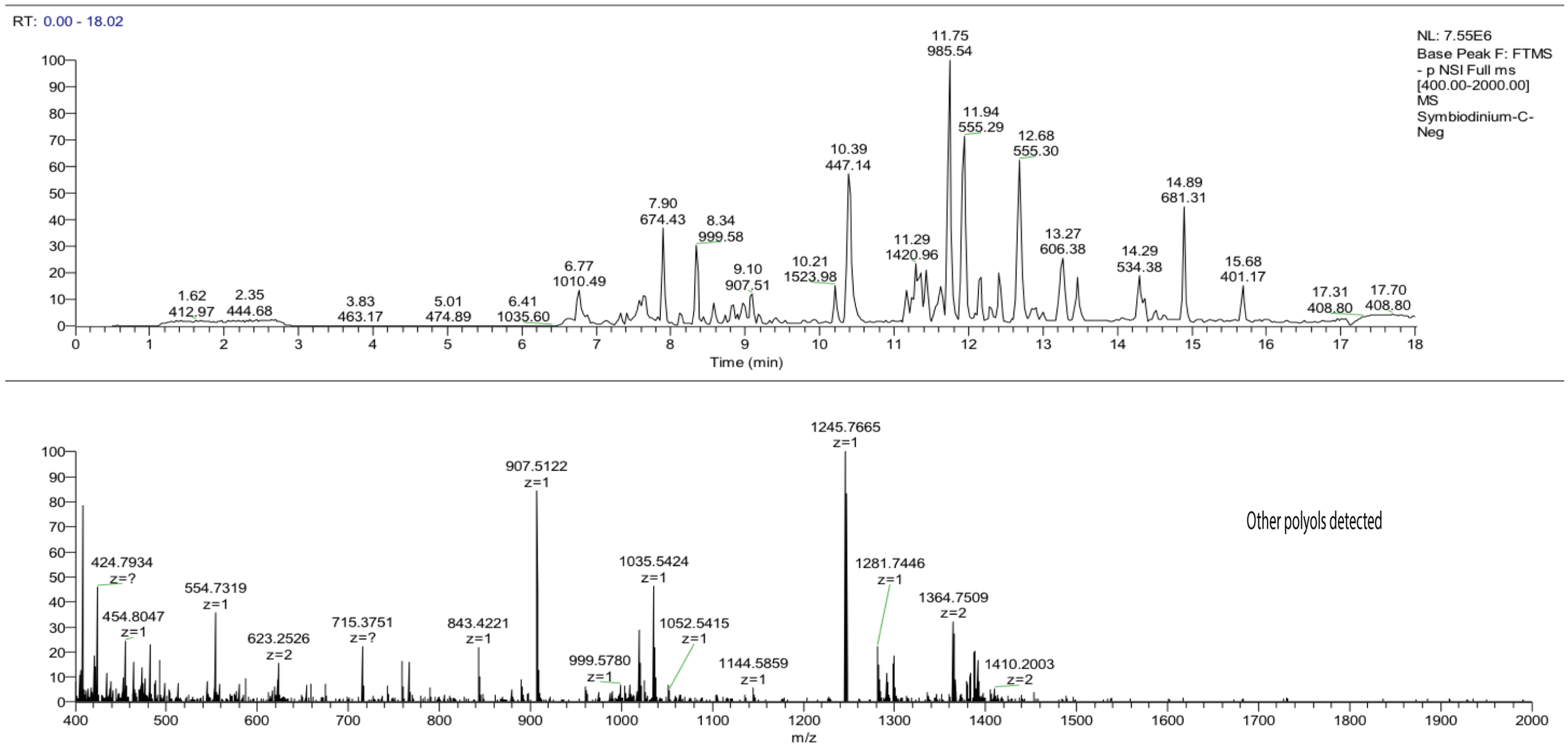

b

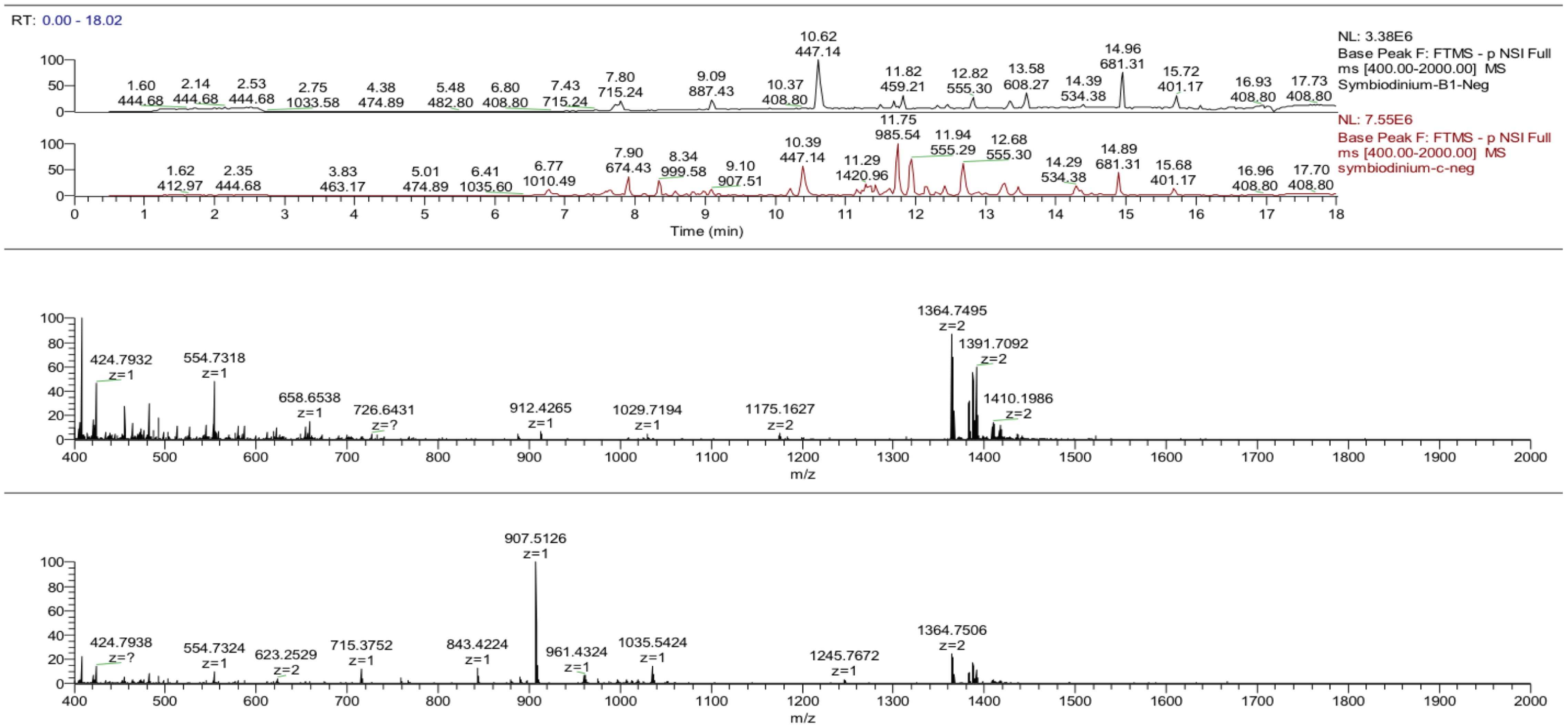

Figure S12: **a.** NanoLC-MS (negative ion) profile and mass spectrum (expanded) of the methanol extract of Clade C. **b.** Similarity of nanoLC-MS (negative ion) profile and mass spectrum (expanded) of the methanol extract of Clade C and B1.

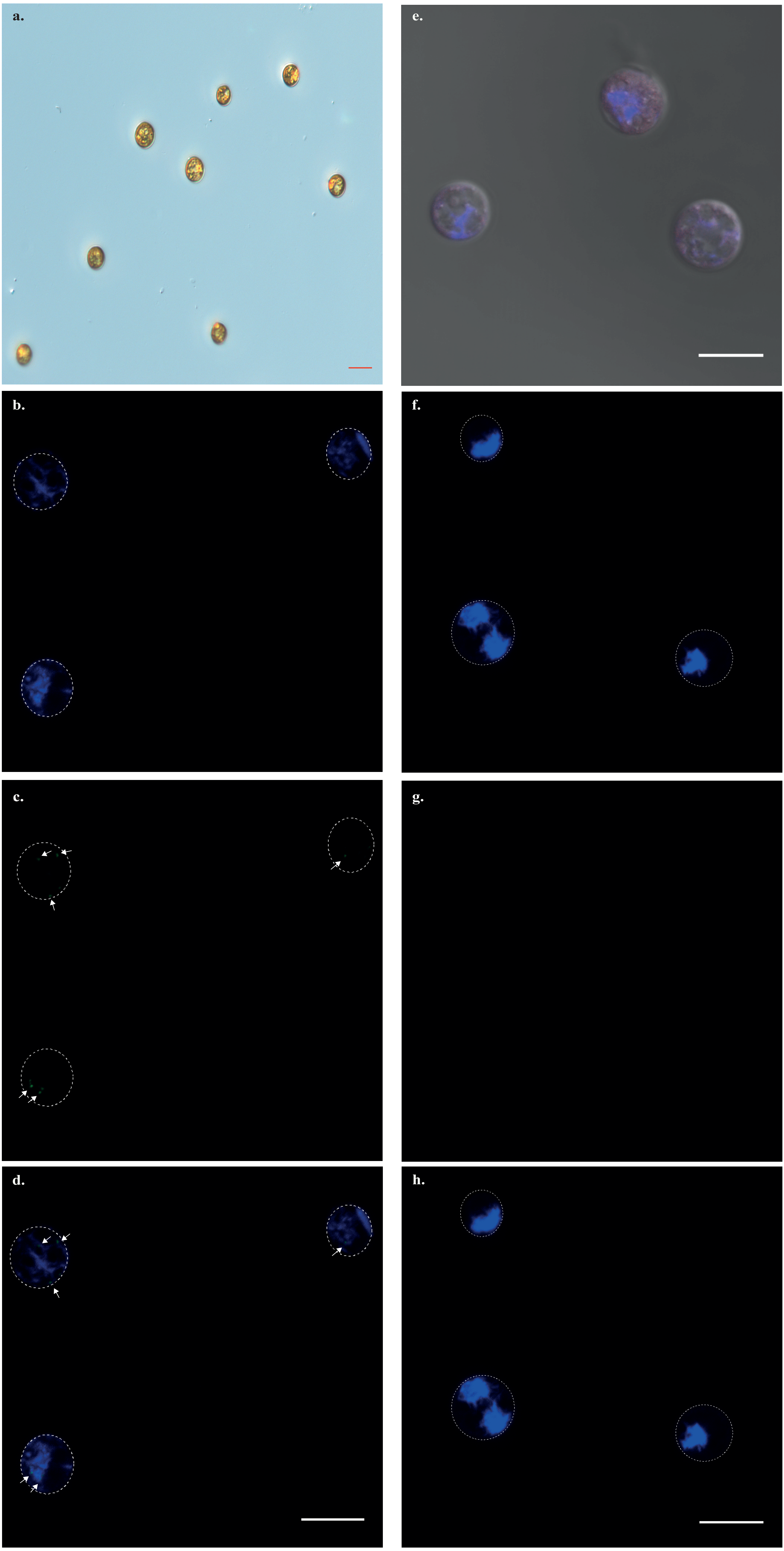

Figure S13. Immunofluorescent staining of Symbiodinium cells with anti-KS antibody. a. Differential interference contrast (DIC) imaging showing the spherical shape and internal complexity of cells at 40x magnification. b-d. Confocal images (63x) of clade C stained with KS antibody show localization of KS proteins (arrows). Nuclei are stained blue with DAPI (b), KS proteins are in green (c) and merged image of nuclei and KS protein staining (d) e. DIC imaging of cells at 63x showing detailed peripheral localization of chloroplasts (red autofluorescence) and nuclei (blue).
f-h. Confocal images (63x) of control cells stained with only secondary antibody. Nuclei are stained with DAPI (f), no KS protein were stained (g) and merged image of nuclei and no-KS staining (h).White dotted lines show the cell outlines. Scale bars are 10 μm in the panels.

Supplementary Table S1: Ketosynthase domain containing genes in *Symbiodinium* clades used in this study

Gene ID (as used in this study)	Total AA	Pfam domain	BLASTP best hit in NCBI database (Accession #) / Organism	E-value	Identities/ Similarities (% AA)	Scaffold #	Assembled transcriptome ID
symbB1.v1.2.015913.t1 (B1015913.t1)	1671	KS	AQS99262.1 *Gambierdiscus polynesiensis*	0	53/69	1171.1	symbB1.comp20639_c0_seq1
symbB1.v1.2.015790.t1 (B1015790.t1)	427	KS	OLQ06784.1 *Symbiodinium microadriaticum*	0	78/86	1186.1	symbB1.comp52059_c0_seq1
symbB1.v1.2.015789.t1 (B1015789.t1)	1216	KR-PP-KS	OLQ06784.1 *Symbiodinium microadriaticum*	0	73/81	1186.1	symbB1.EST_k37c20_822
symbB1.v1.2.017689.t1 (B1017689.t1)	2261	KS-Cupin 8	OLP92465.1 *Symbiodinium microadriaticum*	0	70/80	1368.1	symbB1.EST_k37c20_37774
symbB1.v1.2.019160.t1 (B1019160.t1)	1068	KS	AQS99255.1 *Gambierdiscus polynesiensis*	0	55/69	1555.1	symbB1.EST_k37c20_8679
symbB1.v1.2.002919.t1 (B1002919.t1)	1105	KS	AQS99273.1 *Gambierdiscus excentricus*	0	52/67	160.1	symbB1.EST_k37c20_17447
symbB1.v1.2.020241.t1 (B1020241.t1)	656	KS	AQS99271.1 *Gambierdiscus excentricus*	0	55/67	1693.1	symbB1.EST_k37c20_3634
symbB1.v1.2.022565.t1 (B1022565.t1)	1547	KS-CNBD	OLP97452.1 *Symbiodinium microadriaticum*	0	67/80	2011.1	symbB1.EST_k37c20_6838
symbB1.v1.2.028834.t1 (B1028834.t1)	2431	KS	OLQ02136.1 *Symbiodinium microadriaticum*	0	81/91	3094.1	symbB1.EST_k37c20_17396
symbB1.v1.2.030435.t1 (B1030435.t1)	481	KS-AT	OLP78851.1 *Symbiodinium microadriaticum*	6E-120	59/73	3430.1	symbB1.comp58270_c0_seq1
symbB1.v1.2.036410.t1 (B1036410.t1)	3519	A-KS-KR-PP-KS-DH-KR-PP-KS	OLP85931.1 *Symbiodinium microadriaticum*	0	42/55	5132.1	symbB1.comp56297_c0_seq6
symbB1.v1.2.008781.t1 (B1008781.t1)	2107	DH-KR-PP-PP-KS-DH	OLQ06785.1 *Symbiodinium microadriaticum*	0	80/85	514.1	symbB1.comp69166_c0_seq1
symbB1.v1.2.008782.t1 (B1008782.t1)	1848	KR-PP-KS-KR-PP-KS	OLQ06785.1 *Symbiodinium microadriaticum*	0	75/84	514.1	symbB1.comp53648_c0_seq1
symbB1.v1.2.001928.t1 (B1001928.t1)	1164	KS	AQS99229.1 *Gambierdiscus excentricus*	0	45/60	55.1	symbB1.comp17616_c0_seq1
symbB1.v1.2.001307.t1 (B1001307.t1)	797	KS	OLP77034.1 *Symbiodinium microadriaticum*	0	76/82	57.1	symbB1.EST_k37c20_6869
symbB1.v1.2.037839.t1 (B1037839.t1)	604	KS	OLP95534.1 *Symbiodinium microadriaticum*	0	81/91	5703.1	symbB1.comp5164_c0_seq1
symbB1.v1.2.040026.t1 (B1040026.t1)	582	KS	AFW98411.1 *Alexandrium ostenfeldii*	0	68/81	6945.1	symbB1.comp4031_c0_seq1
symbB1.v1.2.013880.t1 (B1013880.t1)	1071	KS	AQS99258.1 *Gambierdiscus excentricus*	0	40/57	991.1	symbB1.comp24939_c0_seq1
symbB1.v1.2.012436.t1 (B1012436.t1)	10601	See Figure 2 (Beedessee et al., 2015)	–	–	–	860.1	symbB1.comp70898_c0_seq1
symbB1.v1.2.013202.t1 (B1013202.t1)	940	KS	AQS99274.1 Gambierdiscus excentricus	0	51/65	929.1	symbB1.comp10295_c0_seq1
symbB1.v1.2.021684.t1 (B1021684.t1)	805	KS	OLQ02729.1 *Symbiodinium microadriaticum*	0	76/87	1888.1	symbB1.comp7458_c0_seq1
symbB1.v1.2.029496.t1 (B1029496.t1)	4982	VCBS-KS-DH-KR-PP-KS-KR-PP	OLP85322.1 *Symbiodinium microadriaticum*	0	65/75	3236.1	symbB1.comp53836_c0_seq1
symbB1.v1.2.003781.t1 (B1003781.t1)	4222	KS-AT-DH-PP-ER(ADH_zinc)-KR-DNA methylase	*OLQ06785.1* *Symbiodinium microadriaticum*	0	67/78	214.1	symbB1.comp84707_c0_seq1
symbB1.v1.2.017906.t1 (B1017906.t1)	615	KS	OLP82529.1 *Symbiodinium microadriaticum*	0	87/93	1404.1	symbB1.comp2635_c0_seq1
symbB1.v1.2.036344..t1 (B1036344.t1)	722	KS	AQS99221.1 Gambierdiscus polynesiensis	0	46/65	5099.1	symbB1.comp13568_c0_seq1
symbB1.v1.2.027671.t1 (B1027671.t1)	957	A-PP-KS-AT	OLP83938.1 *Symbiodinium microadriaticum*	0	72/79	2857.1	symbB1.EST_k37c20_5813
symbB1.v1.2.036002.t1 (B1036002.t1)	2987	A-PP-KS-KR-DH	OLQ14315.1 *Symbiodinium microadriaticum*	0	55/68	4981.1	symbB1.EST_k37c20_11234

symbB1.v1.2.015788.t2 (B1015788.t2)	920	KR-PP-KS-AT	OLQ06784.1 *Symbiodinium microadriaticum*	0	75/84	1186.1	symbB1.comp40305_c0_seq1
symbB1.v1.2.000535.t1 (B1000535.t1)	4838	KS	OLQ08992.1 *Symbiodinium microadriaticum*	0	42/59	31.1	symbB1.EST_k37c20_11702
s128_g23.t2 (As128_g23.t2)	879	Heme oxygenase-KS-Heme oxygenase	OLQ07573.1 *Symbiodinium microadriaticum*	0	88/88	128.1	comp44668_c0_seq1
s4308_g6.t1 (A s4308_g6.t1)	991	KS	OLP88203.1 *Symbiodinium microadriaticum*	0	99/99	4308.1	comp28303_c0_seq1
s3551_g7.t1 (As3551_g7.t1)	1027	RRM1-RRM1-KS	OLP78656.1 *Symbiodinium microadriaticum*	0	94/95	3551.1	comp8100_c0_seq1
s1303_g11.t1 (As1303_g11.t1)	1353	Pkinase-KS	OLP77034.1 *Symbiodinium microadriaticum*	0	78/80	1303.1	comp29404_c0_seq1
s1906_g6.t1 (As1906_g6.t1)	2244	KS-RAP-Cyt-b5	OLP84449.1 *Symbiodinium microadriaticum*	0	93/93	1906.1	comp41299_c0_seq1
s652_g6.t1 (As652_g6.t1)	813	Ank2-Ank2-Ank2-KS	OLQ02729.1 *Symbiodinium microadriaticum*	0	90/92	652.1	comp39686_c0_seq1
s1087_g8.t1 (As1087_g8.t1)	1902	AAA-MgA_C-KS	AQS99273.1 *Gambierdiscus excentricus*	0	51/67	1087.1	comp34635_c0_seq1
s4302_g9.t2 (As4302_g9.t2)	784	KS	OLP88440.1 *Symbiodinium microadriaticum*	0	98/98	4302.1	comp34198_c0_seq1
s2561_g15.t2 (As2561_g15.t2)	1033	KS	AQS99289.1 *Gambierdiscus excentricus*	0	66/80	2561.1	comp24392_c0_seq2
s7155_g1.t1 (As7155_g1.t1)	1042	KS	OLP92465.1 *Symbiodinium microadriaticum*	0	83/84	7155.1	comp33794_c0_seq1
s3787_g5.t1 (As3787_g5.t1)	1188	KS	OLP97452.1 *Symbiodinium microadriaticum*	0	97/97	3787.1	comp18293_c0_seq1
s1323_g8.t1 (As1323_g8.t1)	1040	KS	AQS99262.1 *Gambierdiscus excentricus*	0	52/69	1323.1	comp6314_c0_seq1
s2786_g5.t4 (As2786_g5.t4)	1245	KS	AQS99286.1 *Gambierdiscus polynesiensis*	0	49/65	2786.1	comp9936_c0_seq1
s572_g26.t3 (As572_g26.t3)	2604	RVT2-Fucokinase-KS	AQS99318.1 *Gambierdiscus polynesiensis*	0	62/76	572.1	comp30690_c0_seq1
s1629_g33.t1 (As1629_g33.t1)	944	KS	OLP88021.1 *Symbiodinium microadriaticum*	0	85/85	1629.1	comp28622_c0_seq1
s5474_g6.t1 (As5474_g6.t1)	1160	KS	AQS99229.1 *Gambierdiscus excentricus*	0	45/60	5474.1	comp31618_c0_seq1
s190_g20.t2 (A s190_g20.t2)	2172	DH-ER(ADH_zinc)-KR-PP-KS-DH	OLQ06784.1 *Symbiodinium microadriaticum*	0	77/85	190.1	comp23579_c1_seq4
s527_g6.t1 (As527_g6.t1)	1671	ER(ADH_zinc)-PP-PP-KS-DH	OLQ06784.1 *Symbiodinium microadriaticum*	0	97/98	527.1	comp23579_c0_seq1
s988_g13.t1 (As988_g13.t1)	4258	KS-DH-PP-KS-KR-PP-KS	OLP85322.1 *Symbiodinium microadriaticum*	0	80/83	988.1	comp25448_c0_seq1
s5992_g1.t1 (As5992_g1.t1)	2066	KR-PP-KS-DH	OLP78851.1 *Symbiodinium microadriaticum*	0	87/90	5992.1	comp8106_c0_seq1
s190_g24.t1 (As190_g24.t1)	964	PP-KS-KR	OLQ06785.1 *Symbiodinium microadriaticum*	0	67/79	190.1	comp73459_c0_seq1
s527_g5.t1 (As527_g5.t1)	3108	PP-KS-KR-PP-KS-KR-PP-KS-DH	OLQ06785.1 *Symbiodinium microadriaticum*	0	88/89	527.1	comp74995_c0_seq1
s508_g32.t1 (As508_g32.t1)	1754	A-KS-TE	OLP95547.1 *Symbiodinium microadriaticum*	0	92/65	508.1	comp27989_c0_seq1
s190_g19.t1 (As190_g19.t1)	3439	KR-PP-KS-KR-PP-KS-DH-ER(ADH_zinc)-KR-PP-TE	OLQ06784.1 *Symbiodinium microadriaticum*	0	84/88	190.1	comp27159_c0_seq1
s527_g8.t1 (As527_g8.t1)	2930	KR-PP-KS-KR-PP-KS-DH-ER(ADH_zinc)-KR	OLQ06784.1 *Symbiodinium microadriaticum*	0	91/92	527.1	comp27159_c0_seq1
s892_g17 (Cs892_g17)	976	KS	OLQ02136.1 *Symbiodinium microadriaticum*	0	82/90	892.1	comp29141_c0_seq1
s809_g5 (Cs809_g5.t1)	2768	RVT2-AAA-MgsA_C-KS	AQS99273.1 *Gambierdiscus excentricus*	0	47/63	809.1	comp43784_c0_seq1
s894_g33.t1 (Cs894_g33.t1)	789	KS	AQS99271.1 *Gambierdiscus excentricus*	0	53/66	894.1	comp27826_c0_seq1
s574_g7.t3 (Cs574_g7.t3)	824	KS	AQS99286.1 *Gambierdiscus polynesiensis*	0	51/66	574.1	comp37107_c0_seq1

s144_g38.t1 (Cs144_g38.t1)	1761	TPR12-TPR12-KS	AIW63286.1 *Azadinium spinosum*	0	63/77	144.1	comp24864_c0_seq1
s2246_g6.t1.t1 (Cs2246_g6.t1.t1)	3744	RVT2-LR-KS	OLQ08682.1 *Symbiodinium microadriaticum*	0	55/67	2246.1	comp46226_c0_seq1
s3847_g4.t1 (Cs3847_g4.t1)	2777	KS-cNMP-cNMP-cNMP-cNMP-cNMP-cNMP-cNMP-cNMP	OLP97452.1 *Symbiodinium microadriaticum*	0	68/81	3847.1	comp14688_c0_seq1
s929_g10.t1 (Cs929_g10.t1)	1029	KS	OLP88203.1 *Symbiodinium microadriaticum*	0	85/92	929.1	comp26470_c0_seq1
s121_g22.t1 (Cs121_g22.t1)	1021	KS	AQS99258.1 *Gambierdiscus excentricus*	0	51/67	121.1	comp26243_c0_seq1
s1154_g4.t1 (Cs1154_g4.t1)	949	KS	AQS99197.1 *Gambierdiscus polynesiensis*	0	58/71	1154.1	comp6775_c0_seq1
s612_g34.t1 (Cs612_g34.t1)	1070	KS	AQS99258.1 *Gambierdiscus excentricus*	0	42/57	612.1	comp34408_c0_seq1
s1817_g11.t1 (Cs1817_g11.t1)	1185	KS-KS	OLP84449.1 *Symbiodinium microadriaticum*	0	66/76	1817.1	comp31553_c0_seq1
s895_g13.t1 (Cs895_g13.t1)	1989	KS-FA_hydroxylase-Cyt-b5	AQS99270.1 *Gambierdiscus excentricus*	0	53/67	895.1	comp31899_c0_seq1
s1080_g21.t1 (Cs1080_g21.t1)	788	KS	AQS99274.1 *Gambierdiscus excentricus*	0	51/65	1080.1	comp26500_c0_seq1
s684_g8.t1 (Cs684_g8.t1)	948	KS	AQS99289.1 *Gambierdiscus excentricus*	0	65/80	684.1	comp23033_c0_seq1
s228_g31.t1 (Cs228_g31.t1)	2293	Calx_beta-ABC_tran-KS	AQS99262.1 *Gambierdiscus polynesiensis*	0	54/70	228.1	comp32811_c0_seq1
s1281_g4.t1 (Cs1281_g4.t1)	3069	ER(ADH_zinc)-PP-PP-KS-DH-KR-PP-KS	OLQ06784.1 *Symbiodinium microadriaticum*	0	78/85	1281.1	comp19708_c0_seq1
s1052_g9.t1 (Cs1052_g9.t1)	2164	DH-ER(ADH-zinc)-PP-KS-DH	OLP78851.1 *Symbiodinium microadriaticum*	0	50/65	1052.1	comp4200_c0_seq1
s1029_g9.t1 (Cs1029_g9.t1)	3843	KR-PP-KS-AT-DH-KR-KS-KR-PP-KS	OLQ06785.1 *Symbiodinium microadriaticum*	0	62/71	1029.1	comp60043_c0_seq1
s357_g50.t1 (Cs357_g50.t1)	3523	KS-DH-KR-PP-KS-KR-PP-KS-DH-TPR12-PP	OLP85322.1 *Symbiodinium microadriaticum*	0	66/75	357.1	comp14008_c0_seq1
s1029_g13.t1 (Cs1029_g13.t1)	1142	KS	OLQ06783.1 *Symbiodinium microadriaticum*	0	73/80	1029.1	comp1496_c1_seq1
s1344_g2.t4 (Cs1344_g2.t4)	3413	A-KR-KS-KR-PP-Hexapep	XP_005847910.1 *Chlorella variabilis*	9E-100	50/65	1344.1	comp3199_c0_seq1
s1074_g13.t1 (Cs1074_g13.t1)	1271	A-PP-KS	OLP83938.1 *Symbiodinium microadriaticum*	0	77/86	1074.1	comp14731_c0_seq1
s193_g13.t1 (Cs193_g13.t1)	1763	A-PP-KS-KR-DH	OLQ14315.1 *Symbiodinium microadriaticum*	0	55/69	193.1	comp32064_c0_seq1
s202_g11.t1 (Cs202_g11.t1)	913	KS-PP	AQS99203.1 *Gambierdiscus excentricus*	4.00E-145	43/57	202.1	comp21095_c0_seq1

CNBD: Cyclic nucleotide-binding domain; AH: Aspartyl/Asparaginyl beta-hydroxylase; A: AMP-binding ; PP: PP-binding ; KR: Ketoreductase ; DH:dehydratase ; ER:Enoylreductase ; MT-methyltransferase

Supplementary Table S2: Acyltransferase domain containing genes in *Symbiodinium* clades used in this study

Gene ID (as used in this study)	Total AA	Pfam domain	BLASTP best hit in NCBI database (Accession #) / Organism	E-value	Identities/ Similarities (% AA)	Scaffold #	Assembled transcriptome ID
symbB1.v1.2.039996.t1 (B1039996.t1)	811	KS-AT-DH	OLQ06785.1 *Symbiodinium microadriaticum*	0	73/84	6924.1	symbB1.comp85342_c0_seq1
symbB1.v1.2.003781.t1 (B1003781.t1)	4222	KS-AT-DH-ER(ADH_zinc)-KR-DNA_methylase	OLQ06785.1 *Symbiodinium microadriaticum*	0	67/78	214.1	symbB1.comp94108_c0_seq1
symbB1.v1.2.012436.t1 (B1012436.t1)	10601	See figure 2	—	—	—	860.1	symbB1.comp70898_c0_seq1
symbB1.v1.2.026318.t1 (B1026318.t1)	512	Ank-AT	OLQ10833.1 *Symbiodinium microadriaticum*	1E-99	50/55	2620.1	symbB1.comp29994_c0_seq1
symbB1.v1.2.012467.t1 (B1012467.t1)	592	AT	OLP88585.1 *Symbiodinium microadriaticum*	0	89/95	858.1	symbB1.comp14991_c0_seq1
symbB1.v1.2.015532.t1 (B1015532.t1)	750	HEAT-AT	OLQ05112.1 *Symbiodinium microadriaticum*	0	66/77	1161.1	symbB1.comp33473_c0_seq1
symbB1.v1.2.020811.t1 (B1020811.t1)	426	—	OLP78475.1 *Symbiodinium microadriaticum*	5E-38	35/49	1768.1	symbB1.comp22872_c0_seq1
symbB1.v1.2.022906.t1 (B1022906.t1)	2678	AT-Pkinase-Cupin_4	OLP80999.1 *Symbiodinium microadriaticum*	3E-137	51/65	2052.1	symbB1.comp62393_c0_seq1
symbB1.v1.2.032358.t1 (B1032358.t1)	474	AT	OLP82995.1 *Symbiodinium microadriaticum*	0	85/93	3874.1	symbB1.comp513_c1_seq1
symbB1.v1.2.018170.t1 (B1018170.t1)	1162	Ank-AT-FKBP_C	OLQ01506.1 *Symbiodinium microadriaticum*	0	67/77	1438.1	symbB1.comp18682_c0_seq1
s2684_g3.t1 (As2684_g3.t1)	1080	AT	OLP78475.1 *Symbiodinium microadriaticum*	0	95/97	2684.1	comp21089_c0_seq2
s41_g7.t1 (As41_g7.t1)	553	AT	OLP87520.1 *Symbiodinium microadriaticum*	0	89/90	41.1	comp30651_c0_seq1
s1206_g16.t1 (As1206_g16.t1)	593	Ank-AT	OLP88585.1 *Symbiodinium microadriaticum*	0	98/98	1206.1	comp28052_c0_seq1
s4164_g5.t1 (As4164_g5.t1)	723	HEAT-HEAT-HEAT-HEAT-AT	OLQ05112.1 *Symbiodinium microadriaticum*	0	89/91	4164.1	comp32220_c0_seq1
s126_g21.t1 (As126_g21.t1)	957	AT	OLP88585.1 *Symbiodinium microadriaticum*	6E-77	33/48	126.1	comp32072_c0_seq1
s7547_g2.t1 (As7547_g2.t1)	545	AT	OLP85020.1 *Symbiodinium microadriaticum*	0	94/98	7547.1	comp46533_c0_seq1
s1018_g3.t1 (As1018_g3.t1)	732	AT	OLQ01506.1 *Symbiodinium microadriaticum*	2E-35	36/56	1018.1	comp20971_c0_seq1
s665_g11.t1 (As665_g11.t1)	342	—	OLP99092.1 *Symbiodinium microadriaticum*	1E-110	88/92	665.1	comp36726_c0_seq1
s3570_g15.t1 (As3570_g15.t1)	601	AT	OLP82995.1 *Symbiodinium microadriaticum*	0	87/87	3570.1	comp27427_c0_seq1
s138_g8.t1 (As138_g8.t1)	612	AT	OLQ01506.1 *Symbiodinium microadriaticum*	0	92/95	138.1	comp58116_c0_seq1
s792_g3.t2 (As792_g3.t2)	535	AT	AQS99288.1 *Gambierdiscus belizeanus*	4E-166	79/87	792.1	comp29039_c0_seq1
s3019_g1.t3 (Cs3019_g1.t3)	1085	KR-PP-KS-AT	AQS99305.1 *Gambierdiscus polynesiensis*	0	58/71	3019.1	comp62915_c0_seq1
s24_g36.t1 (Cs24_g36.t1)	590	AT	OLP87520.1 *Symbiodinium microadriaticum*	0	75/83	24.1	comp24242_c0_seq1
s429_g17.t1 (Cs429_g17.t1)	1920	RRM2-AT	OLP88585.1 *Symbiodinium microadriaticum*	0	88/94	429.1	comp6509_c1_seq1
s2692_g2.t1 (Cs2692_g2.t1)	502	HEAT-AT	OLQ05112.1 *Symbiodinium microadriaticum*	0	71/81	2692.1	comp33492_c0_seq1
s893_g20.t1 (Cs893_g20.t1)	998	AT-DUF3179	OLP77179.1 *Symbiodinium microadriaticum*	0	64/73	893.1	comp33060_c0_seq1
s118_g27.t1 (Cs118_g27.t1)	898	AT	OLP85020.1 *Symbiodinium microadriaticum*	2E-75	74/83	118.1	comp42492_c0_seq1
s1269_g23.t3 (Cs1269_g23.t3)	931	Ion_trans	OLP95654.1 *Symbiodinium microadriaticum*	0	55/66	1269.1	comp7307_c0_seq1

s1989_g18.t1 (Cs1989_g18.t1)	741	AT	OLQ01506.1 *Symbiodinium microadriaticum*	1E-89	54/71	1989.1	comp41384_c0_seq1
s2315_g3.t1 (Cs2315_g3.t1)	446	AT	OLP82995.1 *Symbiodinium microadriaticum*	0	79/88	2315.1	comp22776_c0_seq1
s1441_g2.t1 (Cs1441_g2.t1)	1134	Ank-AT-FKBP_C	OLQ01506.1 *Symbiodinium microadriaticum*	0	69/76	1441.1	comp29346_c0_seq1

Ank: ankyrin repeat ; PP: PP-binding; KR: Ketoreductase ; DH: dehydratase; KS: ketosynthase ; DH:dehydratase ; ER:Enoylreductase

Supplementary Table S3: Condenstion domain containing genes in *Symbiodinium* clades used in this study

**Gene ID (as used in this study)**	**Total AA**	**Pfam domain**	**BLASTP best hit in NCBI database (Accession #) / Organism**	**E-value**	**Identities/ Similarities (% AA)**	**Scaffold #**	**Assembled transcriptome ID**
s451_g5.t4 (As451_g5.t4)	2455	tRNA-synt2-A-PP-C	OLP93967.1 *Symbiodinium microadriaticum*	0	92/94	451.1	comp28462_c0_seq1
s5334_g1.t7 (As5334_g1.t7)	2613	A-PP-C-A-PP-NAD_binding4	BAZ14320.1 *Calothrix sp. NIES-4071*	1E-151	35/53	5334.1	comp26380_c0_seq1
symbB1.v1.2.012436.t1 (B1012436.t1)	10601	See Figure 2 (Beedessee et al., 2015)	–	–	–	860.1	symbB1.comp70898_c0_seq1
symbB1.v1.2.036245.t1 (B1036245.t1)	1688	A-PP-C-A	SFJ64597.1 *Thermoflavimicrobium dichotomicum*	0	31/48	5072.1	symbB1.comp61452_c0_seq1
symbB1.v1.2.038923.t1 (B038923.t1)	1424	A-PP-C-A	BAZ14320.1 *Calothrix sp. NIES-4071*	7E-148	36/53	6242.1	symbB1.comp49186_c0_seq1
s535_g6.t1 (Cs535_g6.t1)	1661	C-Ank-Ank-Ank-Ank-Ank	OLP79889.1 *Symbiodinium microadriaticum*	0	86/93	535.1	comp20892_c1_seq1

Ank: ankyrin repeat ; A: AMP-binding ; C: condensation ; PP: PP-binding

Supplementary Table S4: Adenylation domain containing genes in *Symbiodinium* clades used in this study

Gene ID (as used in this study)	Total AA	Pfam domain	BLASTP best hit in NCBI database (Accession #) / Organism	E-value	Identities/ Similarities (% AA)	Scaffold #	Assembled transcriptome ID
s112_g4.t1 (As112_g4.t1)	1489	A-SAS_6-Pkinase	OLP94465.1 *Symbiodinium microadriaticum*	0	83/85	112.1	comp36390_c0_seq1
s1249_g3.t2 (As1249_g3.t2)	1186	A-DUF1796	OLQ07141.1 *Symbiodinium microadriaticum*	0	77/80	1249.1	comp19601_c0_seq1
s1272_g6.t1 (As1272_g6.t1)	1176	A	OLP80648.1 *Symbiodinium microadriaticum*	0	92/93	1272.1	comp45077_c0_seq1
s1794_g4.t1 (As1794_g4.t1)	2175	A-MT_31-PP-TE	OLQ01805.1 *Symbiodinium microadriaticum*	0	94/95	1794.1	comp36046_c0_seq1
s1249_g3.t2 (As1249_g3.t2)	2400	A-Thioredoxin_8-A-MT_24	OLP92825.1 *Symbiodinium microadriaticum*	1E-151	58/60	1977.1	comp33669_c0_seq1
s2138_g2.t1 (As2138_g2.t1)	1009	A	OLQ05334.1 *Symbiodinium microadriaticum*	0	95/96	2138.1	comp7456_c0_seq1
As3629_g2.t1 (As3629_g2.t1)	708	A-PP	OLP84418.1 *Symbiodinium microadriaticum*	0	91/94	3629.1	comp23816_c0_seq1
As451_g6.t3 (As451_g6.t3)	3368	A-PP-C-A-PP-TE-zf_UBR	WP_010249951.1 *Acetivibrio cellulolyticus*	0	30/47	451.1	comp20515_c0_seq1
s5334_g1.t7 (As5334_g1.t7)	2613	A-PP-C-A-PP-NAD_binding4	BAZ14320.1 *Calothrix sp. NIES-4071*	1E-151	35/53	5334.1	comp26380_c0_seq1
As6285_g1.t1 (As6285_g1.t1)	1215	A	OLP79924.1 *Symbiodinium microadriaticum*	0	92/94	6285.1	comp32724_c0_seq1
As745_g3.t1 (As745_g3.t1)	1735	A-PP	OLP85140.1 *Symbiodinium microadriaticum*	0	68/77	745.1	comp26832_c1_seq1
symbB1.v1.2.012436.t1 (B1012436.t1)	10601	See Figure 2 (Beedessee et al., 2015)	–	–	–	860.1	symbB1.comp70898_c0_seq1
symbB1.v1.2.012575.t2 (B1012575.t2)	1893	A-Ank	OLQ14049.1 *Symbiodinium microadriaticum*	0	71/79	851.1	symbB1.comp40116_c0_seq1
symbB1.v1.2.016581.t1 (B1016581.t1)	1169	A	WP_012411748.1 *Nostoc punctiforme*	6E-65	32/49	1253.1	symbB1.comp14532_c0_seq1
symbB1.v1.2.024196.t1 (B1024196.t1)	1018	A	OLP94465.1 *Symbiodinium microadriaticum*	0	45/58	2271.1	symbB1.comp8955_c0_seq1
symbB1.v1.2.027502.t1 (B1027502.t1)	1014	A-PP-C	WP_069865021.1 *Pseudomonas sp. CCA 1*	6E-94	33/51	2828.1	symbB1.comp65743_c0_seq1
symbB1.v1.2.030374.t1 (B1030374.t1)	1687	PseudoU_synth-A-DUF1796-ATP_grasp	OLP94465.1 *Symbiodinium microadriaticum*	7E-134	83/91	3260.1	symbB1.comp61879_c0_seq1
symbB1.v1.2.031573.t1 (B1031573.t1)	1765	A-PP-zf_CCHC	OLP85280.1 *Symbiodinium microadriaticum*	0	70/80	3629.1	symbB1.comp17330_c0_seq1
symbB1.v1.2.033616.t1 (B1033616.t1)	1626	A-PP-A	OLP85280.1 *Symbiodinium microadriaticum*	0	59/69	4203.1	symbB1.comp1062_c0_seq1
symbB1.v1.2.034688.t1 (B1034688.t1)	1028	A	OLP94465.1 *Symbiodinium microadriaticum*	0	47/61	4523.1	symbB1.comp15044_c0_seq1
symbB1.v1.2.034833.t1 (B1034833.t1)	4184	A-RVT2-PP-C-A-TE	ABA73589.1 *Pseudomonas fluorescens Pf0-1*	9E-128	30/47	4570.1	symbB1.comp56548_c0_seq1
symbB1.v1.2.036245.t1 (B1036245.t1)	1688	A-PP-C-A	SFJ64597.1 *Thermoflavimicrobium dichotomicum*	0	31/48	5072.1	symbB1.comp61452_c0_seq1
symbB1.v1.2.039731.t1 (B1039731.t1)	1496	A	OLQ01805.1 *Symbiodinium microadriaticum*	0	72/81	6749.1	symbB1.comp15708_c0_seq1
s1074_g13.t3 (Cs1074_g13.t3)	1473	A-PP-KS	OLP83938.1 *Symbiodinium microadriaticum*	0	77/86	1074.1	comp14731_c0_seq1
s115_g25.t1 (Cs115_g25.t1)	1120	A-A-PP	OLP85280.1 *Symbiodinium microadriaticum*	0	61/72	115.1	comp31049_c0_seq1
s1214_g6.t3 (Cs1214_g6.t3)	962	A-PP-Ank-Ank	OLQ14049.1 *Symbiodinium microadriaticum*	0	63/72	1214.1	comp11808_c0_seq1
s193_g13.t1 (Cs193_g13.t1)	1763	A-PP-KS-KR-DH	OLQ14315.1 *Symbiodinium microadriaticum*	0	55/69	193.1	comp32064_c0_seq1
s2146_g10.t1 (Cs2146_g10.t1)	1232	A-PP-DUF1796	OLQ07141.1 *Symbiodinium microadriaticum*	0	41/54	2146.1	comp16423_c0_seq1
s2227_g13.t2 (Cs2227_g13.t2)	1644	A-PP	OLQ07141.1 *Symbiodinium microadriaticum*	0	47/61	2227.1	comp3247_c0_seq1
s227_g29.t1 (Cs227_g29.t1)	1212	PseudoU_synth-A	OLQ07141.1 *Symbiodinium microadriaticum*	3E-160	68/78	227.1	comp21373_c0_seq1

s2601_g7.t1 (Cs2601_g7.t1)	1090	A	WP_011613194.1 *Trichodesmium erythraeum*	9E-128	45/60	2601.1	comp6114_c1_seq1
s268_g7.t1 (Cs268_g7.t1)	1195	A	OKH30812.1 *Nostoc calcicola FACHB-389*	5E-59	33/48	268.1	comp16997_c0_seq2
s3964_g1.t1 (Cs3964_g1.t1)	1412	A	OLP85140.1 *Symbiodinium microadriaticum*	0	66/75	3964.1	comp10752_c0_seq1
s4353_g5.t1 (Cs4353_g5.t1)	1185	A-PP-C	WP_081001564.1 *Pseudomonas chlororaphis*	1E-106	31/48	4353.1	comp21830_c1_seq1
s830_g19.t1 (Cs830_g19.t1)	1642	A-MT_31-PP-TE	OLP85140.1 *Symbiodinium microadriaticum*	0	71/80	830.1	comp23784_c0_seq1

C: Condensation; Ank:Anykyrin repeat; A: AMP-binding ; PP: PP-binding ; KR: Ketoreductase ; DH:dehydratase;TE:Thioesterase; MT-methyltransferase

Supplementary Table S5: Prediction scores of ChloroP and TargetP, supported by DeepLoc subcellular localization predictor

	Gene model	ChloroP (cTP)	CS-score	cTP-length	TargetP (NN scores)	Location	RC	DeepLoc Location prediction
(a) KS domains from Fig. 1	As190_g19.t1				0.81	M	3	0.6096(peroxisome)
	As988_g13.t1				0.851	M	2	0.4082(peroxisome)
	As4308_g6.t1	0.521	6.483	49				0.4456(plastid)
	A572_g26.t3	0.524	3.138	37				0.6408(plastid)
	As1323_g8.t1				0.591	M	5	0.4421(mitochondrion)
	B1003781.t1				0.972	S	1	0.4811 (peroxisome)
	B1028834.t1				0.93	S	1	0.243(cytoplasm)
	B1007906.t1				0.698	S	5	0.5762(peroxisome)
	B1013880.t1				0.815	M	2	0.6716 (peroxisome)
	b1013202.t1				0.509	S	4	0.2777 (mitochondrion)
	Cs894_g33.t1				0.832	S	2	0.4855 (mitotochondrion)
	Cs1029_g13.t1				0.994	S	1	0.2596 (extracellular)
	Cs202_g11.t1.t7	0.508	4.13	72				0.3274 (plastid)
(b) AT domains from Fig. 2	Cs2692_g2.t11	0.519	11.239	46				0.367 (plastid)
	B1015532.t1				0.741	M	5	0.5208 (cytoplasm)
	B1012467.t1				0.661	M	3	0.6246 (mitochondrion)

cTP tells whether or not this is predicted as a cTP-containing sequence.

CS-score is the MEME scoring matrix score for the suggested cleavage site according to ChloroP.

cTP-length is the predicted length of the presequence

NN scores on which the final prediction of TargetP is based and the location with the highest score is the most likely.

RC is the reliability class, from 1 to 5, where 1 indicates the strongest prediction.

Supplementary Table S6: Features of LTR-retrotransposons identified from PKS-associated scaffolds studied in this paper.

Clade	Scaffold #	Location	Length	Strand	Score[a]	5'-LTR[b]	3'-LTR[c]	PBS (Primer Binding Sites)[d]	PPT (Polypurine tract)[e]
B1	1171.1	95013 - 98077	3065	−	6	95013 - 95130 Len: 118	97960 - 98077 Len: 118	[14/17] 97872 - 97888 (-AlaCGC)	[11/15] 95168 - 95182
	2011.1	23 - 6079	6057	−	6	23 - 173 Len: 151	5929 - 6079 Len: 151	[14/17] 5908 - 5924 (-GlnCTG)	[13/15] 174 - 188
	5132.1	18094 - 25320	7227	+	7	18094 - 19213 Len: 1120	24159 - 25320 Len: 1162	[15/18] 19282 - 19299 (MetCAT)	[11/15] 24111 - 24125
	55.1	181361 - 187363	6003	−	7	181361 - 181472 Len: 112	187250 - 187363 Len: 114	[15/21] 187188 - 187208 (-IleTAT)	[11/15] 181516 - 181530
	57.1	96596 - 100499	3904	−	6	96596 - 97893 Len: 1298	99189 - 100499 Len: 1311	[15/25] 99098 - 99122 (-MetCAT)	[12/15] 97949 - 97963
	991.1	10007 - 16876	6870	−	6	10007 - 10738 Len: 732	16156 - 16876 Len: 721	[15/18] 16068 - 16085 (-GlnCTG)	[11/15] 10816 - 10830
		13136 - 16374	3239	+	6	13136 - 13346 Len: 211	16179 - 16374 Len: 196	[14/19] 13417 - 13435 (LeuCAG)	[12/15] 16164 - 16178
		13392 - 16876	3485	−	6	13392 - 13882 Len: 491	16391 - 16876 Len: 486	[16/24] 16359 - 16382 (-SerCGA)	[11/15] 13960 - 13974
		110819 - 118941	8123	+	7	110819 - 110926 Len: 108	118834 - 118941 Len: 108	[14/19] 110973 - 110991 (GlnCTG)	[12/15] 118750 - 118764
	214.1	10996 - 23070	12075	+	6	10996 - 11125 Len: 130	22959 - 23070 Len: 112	[15/19] 11203 - 11221 (AlaTGC)	[12/15] 22944 - 22958
		233839 - 242959	9121	−	7	233839 - 233994 Len: 156	242804 - 242959 Len: 156	[15/19] 242763 - 242781 (-LeuTAG)	[11/15] 233995 - 234009
	5099.1	19008 - 25190	6183	−	6	19008 - 19160 Len: 153	25024 - 25190 Len: 167	[15/20] 24990 - 25009 (-SerCGA)	[13/15] 19235 - 19249
	214.1	10996 - 23070	12075	+	6	10996 - 11125 Len: 130	22959 - 23070 Len: 112	[15/19] 11203 - 11221 (AlaTGC)	[12/15] 22944 - 22958
		233839 - 242959	9121	−	8	233839 - 233994 Len: 156	242804 - 242959 Len: 156	[15/19] 242763 - 242781 (-LeuTAG)	[11/15] 233995 - 234009
	2052.1	52858 - 62137	9280	−	6	52858 - 52960 Len: 103	62036 - 62137 Len: 102	[15/20] 61936 - 61955 (-AspGTC)	[11/15] 52966 - 52980
	31.1	384910 - 392457	7548	−	6	384910 - 385045 Len: 136	392322 - 392457 Len: 136	[14/21] 392224 - 392244 (-SerAGA)	[12/15] 385118 - 385132
A3	3551.1	22815 - 26576	3762	−	6	22815 - 22937 Len: 123	26463 - 26576 Len: 114	ND	[13/15] 22997 - 23011
		25224 - 39923	14700	−	6	25224 - 25331 Len: 108	39818 - 39923 Len: 106	ND	[11/15] 25347 - 25361
		25824 - 44954	19131	−	6.5	25824 - 26076 Len: 253	44706 - 44954 Len: 249	[14/19] 44659 - 44677 (-SerCGA)	[11/15] 26080 - 26094
		25826 - 45437	19612	−	6	25826 - 26560 Len: 735	44708 - 45437 Len: 730	[14/19] 44659 - 44677 (-SerCGA)	[12/15] 26578 - 26592
		27416 - 46553	19138	−	6	27416 - 28184 Len: 769	45798 - 46553 Len: 756	ND	[14/15] 28202 - 28216
		38907 - 44166	5260	+	6	38907 - 39910 Len: 1004	43214 - 44166 Len: 953	[14/18] 39930 - 39947 (LeuTAG)	[12/15] 43138 - 43152
		39072 - 43376	4305	+	6	39072 - 39185 Len: 114	43264 - 43376 Len: 113		[11/15] 43191 - 43205
		39698 - 49594	9897	+	6	39698 - 39910 Len: 213	49382 - 49594 Len: 213	[14/18] 39930 - 39947 (LeuTAG)	[12/15] 49300 - 49314
		40452 - 44835	4384	−	6	40452 - 40576 Len: 125	44711 - 44835 Len: 125	[14/19] 44659 - 44677 (-SerCGA)	[11/15] 40610 - 40624
		43228 - 45094	1867	−	6	43228 - 43559 Len: 332	44751 - 45094 Len: 344	[14/19] 44659 - 44677 (-SerCGA)	[12/15] 43593 - 43607
		44734 - 50473	5740	+	6	44734 - 44938 Len: 205	50269 - 50473 Len: 205	ND	[12/15] 50240 - 50254
	2561.1	45561 - 57214	11654	−	6	45561 - 46004 Len: 444	56768 - 57214 Len: 447	ND	[11/15] 46067 - 46081
	572.1	40146 - 44099	3954	+	6	40146 - 40852 Len: 707	43402 - 44099 Len: 698	[14/21] 40904 - 40924 (SerAGA)	[14/15] 43366 - 43380
		42198 - 46411	4214	+	6	42198 - 43173 Len: 976	45414 - 46411 Len: 998	[14/18] 43177 - 43194 (LeuTAG)	[11/15] 45399 - 45413
		42652 - 46411	3760	+	7	42652 - 43173 Len: 522	45907 - 46411 Len: 505	[14/18] 43177 - 43194 (LeuTAG)	[13/15] 45881 - 45895

		229591 - 231955	2365	−	6	229591 - 229821 Len: 231	231739 - 231955 Len: 217	[14/19] 231658 - 231676 (-MetCAT)	ND
		229591 - 231955	2365	−	6	229591 - 230049 Len: 459	231505 - 231955 Len: 451	[14/19] 231424 - 231442 (-MetCAT)	ND
		229591 - 231955	2365	−	6	229591 - 229935 Len: 345	231625 - 231955 Len: 331	[14/19] 231538 - 231556 (-MetCAT)	ND
		229591 - 231955	2366	−	7	229591 - 230106 Len: 516	231448 - 231955 Len: 508	[14/20] 231376 - 231395 (-MetCAT)	ND
	1629.1	70450 - 79049	8600	−	6	70450 - 70720 Len: 271	78779 - 79049 Len: 271	ND	[13/15] 70768 - 70782
		241976 - 251263	9288	+	6	241976 - 242420 Len: 445	250808 - 251263 Len: 456	ND	[12/15] 250728 - 250742
	190.1	144382 - 146079	1698	+	6	144382 - 144603 Len: 222	145857 - 146079 Len: 223	[14/20] 144647 - 144666 (GlnCTG)	ND
		213011 - 215482	2472	+	6	213011 - 213143 Len: 133	215343 - 215482 Len: 140	[14/20] 213222 - 213241 (LeuAAG)	[11/15] 215266 - 215280
		213011 - 215482	2472	+	6	213011 - 213143 Len: 133	215346 - 215482 Len: 137	[14/20] 213222 - 213241 (LeuAAG)	[11/15] 215266 - 215280
		213059 - 215446	2388	+	6	213059 - 213166 Len: 108	215330 - 215446 Len: 117	[14/20] 213222 - 213241 (LeuAAG)	[11/15] 215266 - 215280
		213059 - 215446	2388	+	6	213059 - 213166 Len: 108	215343 - 215446 Len: 104	[14/20] 213222 - 213241 (LeuAAG)	[11/15] 215266 - 215280
		213059 - 215452	2394	+	6	213059 - 213166 Len: 108	215343 - 215452 Len: 110	[14/20] 213222 - 213241 (LeuAAG)	[11/15] 215266 - 215280
		276622 - 277917	1296	+	7	276622 - 276762 Len: 141	277783 - 277917 Len: 135	[15/21] 276805 - 276825 (LeuAAG)	[12/15] 277733 - 277747
	41.1	96440 - 112431	15992	+	6	96440 - 96548 Len: 109	112325 - 112431 Len: 107	[14/18] 96623 - 96640 (ProTGG)	[11/15] 112293 - 112307
		276004 - 284536	8533	−	6.5	276004 - 276211 Len: 208	284327 - 284536 Len: 210	[17/26] 284291 - 284316 (-ThrCGT)	[12/15] 276223 - 276237
		330216 - 339229	9014	−	6	330216 - 330329 Len: 114	339115 - 339229 Len: 115	[14/19] 339047 - 339065 (-LeuTAG)	[11/15] 330374 - 330388
		399839 - 410874	11036	−	6	399839 - 399942 Len: 104	410771 - 410874 Len: 104	[14/16] 410694 - 410709 (-IleAAT)	[11/15] 399955 - 399969
	1206.1	56613 - 58066	1454	−	6	56613 - 56721 Len: 109	57958 - 58066 Len: 109	[15/19] 57931 - 57949 (-GlnCTG)	[12/15] 56768 - 56782
		56728 - 58457	1730	−	6	56728 - 56840 Len: 113	58345 - 58457 Len: 113	[14/19] 58278 - 58296 (-LeuTAA)	[12/15] 56856 - 56870
	4164.1	59417 - 61467	2051	+	6	59417 - 59730 Len: 314	61142 - 61467 Len: 326	[14/18] 59775 - 59792 (ThrTGT)	[12/15] 61075 - 61089
		61027 - 62264	1238	+	6	61027 - 61137 Len: 111	62154 - 62264 Len: 111	[14/16] 61164 - 61179 (SerTGA)	[12/15] 62082 - 62096
	126.1	72758 - 79309	6552	+	7	72758 - 72931 Len: 174	79134 - 79309 Len: 176	[16/21] 72971 - 72991 (MetCAT)	[14/15] 79091 - 79105
		124617 - 126040	1424	+	6	124617 - 124803 Len: 187	125885 - 126040 Len: 156	[14/19] 124817 - 124835 (TyrGTA)	[12/15] 125844 - 125858
		124694 - 126040	1347	+	6	124694 - 124803 Len: 110	125933 - 126040 Len: 108	[14/19] 124817 - 124835 (TyrGTA)	[12/15] 125844 - 125858
	1018.1	132419 - 152551	20133	−	6	132419 - 132526 Len: 108	152444 - 152551 Len: 108	[14/22] 152344 - 152365 (-SerCGA)	[11/15] 132536 - 132550
		153216 - 158216	5001	−	6	153216 - 153342 Len: 127	158106 - 158216 Len: 111	ND	[11/15] 153345 - 153359
		156331 - 167768	11438	−	6	156331 - 156457 Len: 127	167643 - 167768 Len: 126	[14/20] 167546 - 167565 (-AlaCGC)	ND
		160683 - 177505	16823	−	6	160683 - 160790 Len: 108	177398 - 177505 Len: 108	[14/17] 177316 - 177332 (-GlnCTG)	[12/15] 160854 - 160868
	665.1	7795 - 24932	17138	−	6	7795 - 7898 Len: 104	24796 - 24932 Len: 137	[14/18] 24706 - 24723 (-MetCAT)	[11/15] 7945 - 7959
	138.1	159246 - 164831	5586	+	6	159246 - 159740 Len: 495	164337 - 164831 Len: 495	[14/18] 159802 - 159819 (LeuTAA)	[12/15] 164242 - 164256
	792.1	31964 - 34502	2539	+	6	31964 - 32077 Len: 114	34391 - 34502 Len: 112	[15/20] 32114 - 32133 (LeuTAG)	ND
		98119 - 110925	12807	−	6	98119 - 98439 Len: 321	110597 - 110925 Len: 329	[14/19] 110569 - 110587 (-GluTTC)	[12/15] 98440 - 98454
		98119 - 111134	13016	−	6	98119 - 98665 Len: 547	110597 - 111134 Len: 538	[14/19] 110569 - 110587 (-GluTTC)	[11/15] 98666 - 98680
	527.1	225586 - 227135	1550	+	6	225586 - 225718 Len: 133	227003 - 227135 Len: 133	ND	[12/15] 226949 - 226963
	508.1	80141 - 88969	8829	+	7	80141 - 80378 Len: 238	88732 - 88969 Len: 238	[14/18] 80408 - 80425 (SerAGA)	[12/15] 88678 - 88692
		120408 - 128933	8526	−	6	120408 - 120508 Len: 101	128833 - 128933 Len: 101	[16/20] 128779 - 128798 (-PheGAA)	[11/15] 120509 - 120523
		120408 - 129053	8646	+	6	120408 - 120508 Len: 101	128953 - 129053 Len: 101	[14/17] 120544 - 120560 (AspGTC)	[11/15] 128886 - 128900

		128938 - 137665	8728	−	7	128938 - 129053 Len: 116	137545 - 137665 Len: 121	[16/20] 137511 - 137530 (-PheGAA)	[11/15] 129054 - 129068
		129137 - 137810	8674	+	6	129137 - 129251 Len: 115	137696 - 137810 Len: 115	[14/17] 129276 - 129292 (AspGTC)	[11/15] 137618 - 137632
		183849 - 193303	9455	+	6	183849 - 184136 Len: 288	193020 - 193303 Len: 284	[14/18] 184195 - 184212 (SerTGA)	ND
**C**	892.1	6622 - 26025	19404	−	6	6622 - 6881 Len: 260	25689 - 26025 Len: 337	[14/18] 25603 - 25620 (-IleTAT)	[13/15] 6966 - 6980
		7371 - 16651	9281	+	6	7371 - 8903 Len: 1533	15110 - 16651 Len: 1542	ND	[12/15] 15016 - 15030
		32386 - 41575	9190	−	6	32386 - 32552 Len: 167	41413 - 41575 Len: 163	[15/20] 41325 - 41344 (-SerCGA)	[12/15] 32628 - 32642
		76453 - 84886	8434	−	6	76453 - 76627 Len: 175	84689 - 84886 Len: 198	[14/18] 84650 - 84667 (-GlnCTG)	ND
	894.1	60142 - 63238	3097	−	6	60142 - 60655 Len: 514	62741 - 63238 Len: 498	ND	[13/15] 60685 - 60699
		60142 - 63304	3163	−	6	60142 - 60655 Len: 514	62807 - 63304 Len: 498	ND	[13/15] 60685 - 60699
		84093 - 107701	23609	−	6	84093 - 86248 Len: 2156	105526 - 107701 Len: 2176	ND	[14/15] 86282 - 86296
		86137 - 97800	11664	−	6	86137 - 86248 Len: 112	97689 - 97800 Len: 112	ND	[14/15] 86282 - 86296
		103043 - 104868	1826	−	6	103043 - 103196 Len: 154	104716 - 104868 Len: 153	[14/17] 104649 - 104665 (-GlnTTG)	ND
		254187 - 255899	1713	+	6	254187 - 254467 Len: 281	255618 - 255899 Len: 282	ND	[13/15] 255584 - 255598
		254187 - 257103	2917	+	6	254187 - 254370 Len: 184	256919 - 257103 Len: 185	ND	[13/15] 256885 - 256899
		254191 - 256389	2199	+	6	254191 - 254643 Len: 453	255910 - 256389 Len: 480	ND	[13/15] 255872 - 255886
		254191 - 256389	2199	+	6	254191 - 254741 Len: 551	255816 - 256389 Len: 574	ND	[14/15] 255775 - 255789
	574.1	197963 - 199919	1957	+	6	197963 - 198067 Len: 105	199815 - 199919 Len: 105	[14/19] 198130 - 198148 (SerCGA)	[12/15] 199748 - 199762
		229375 - 232257	2883	+	6.5	229375 - 229583 Len: 209	232040 - 232257 Len: 218	[16/20] 231962 - 231981 (-GlnTTG)	[11/15] 229604 - 229618
		237751 - 239203	1453	−	6	237751 - 237899 Len: 149	239055 - 239203 Len: 149	[14/20] 239003 - 239022 (-GluCTC)	[13/15] 237939 - 237953
	144.1*	71526 - 79598	8073	−	6	71526 - 71719 Len: 194	79410 - 79598 Len: 189	[15/21] 79321 - 79341 (-LeuTAG)	[13/15] 71735 - 71749
		209207 - 211111	1905	+	6	209207 - 209330 Len: 124	210989 - 211111 Len: 123	[15/21] 209408 - 209428 (AlaTGC)	[11/15] 210959 - 210973
		209250 - 211111	1862	+	6	209250 - 209393 Len: 144	210968 - 211111 Len: 144	[15/21] 209408 - 209428 (AlaTGC)	[11/15] 210938 - 210952
		209250 - 211111	1862	+	6	209250 - 209372 Len: 123	210968 - 211111 Len: 144	[15/21] 209408 - 209428 (AlaTGC)	[11/15] 210938 - 210952
		464327 - 466467	2141	+	6	464327 - 464439 Len: 113	466355 - 466467 Len: 113	[16/23] 464483 - 464505 (SerCGA)	[11/15] 466340 - 466354
		464472 - 465948	1477	−	6	464472 - 464629 Len: 158	465789 - 465948 Len: 160	[14/21] 465707 - 465727 (-SerTGA)	[11/15] 464646 - 464660
		527399 - 530149	2751	+	6	527399 - 527896 Len: 498	529669 - 530149 Len: 481	[15/22] 527975 - 527996 (GlnCTG)	[13/15] 529580 - 529594
		527399 - 530158	2760	+	6	527399 - 527905 Len: 507	529669 - 530158 Len: 490	[15/22] 527975 - 527996 (GlnCTG)	[13/15] 529580 - 529594
	2246.1	113 - 2512	2400	+	6	113 - 385 Len: 273	2219 - 2512 Len: 294	[14/18] 441 - 458 (TyrGTA)	[11/15] 2176 - 2190
		527 - 2646	2120	−	6	527 - 635 Len: 109	2538 - 2646 Len: 109	[14/20] 2479 - 2498 (-LeuTAA)	[11/15] 673 - 687
	929.1	121850 - 123407	1558	+	6	121850 - 121967 Len: 118	123283 - 123407 Len: 125	[14/23] 122024 - 122046 (GlnCTG)	ND
		233694 - 235328	1635	+	7	233694 - 233845 Len: 152	235177 - 235328 Len: 152	[14/19] 233906 - 233924 (AsnGTT)	[11/15] 235134 - 235148
	121.1	91055 - 104339	13285	+	6	91055 - 91247 Len: 193	104147 - 104339 Len: 193	[16/22] 91319 - 91340 (SerCGA)	[13/15] 104096 - 104110
		115223 - 125158	9936	−	6	115223 - 115379 Len: 157	124998 - 125158 Len: 161	[14/18] 124898 - 124915 (-SerTGA)	[13/15] 115388 - 115402
		116875 - 132473	15599	+	7	116875 - 117139 Len: 265	132206 - 132473 Len: 268	[15/20] 117219 - 117238 (GlnCTG)	[12/15] 132144 - 132158
		117902 - 126724	8823	−	6	117902 - 118042 Len: 141	126577 - 126724 Len: 148	[14/20] 126506 - 126525 (-LeuCAG)	[11/15] 118082 - 118096
		121024 - 122569	1546	+	6	121024 - 121272 Len: 249	122321 - 122569 Len: 249	[14/19] 121335 - 121353 (MetCAT)	ND

		127000 - 133770	6771	−	6	127000 - 127177 Len: 178	133593 - 133770 Len: 178	[14/18] 133563 - 133580 (-TyrGTA)	[12/15] 127215 - 127229
		198329 - 210153	11825	−	6	198329 - 198436 Len: 108	210044 - 210153 Len: 110	[14/16] 210030 - 210045 (-ValAAC|ValTAC)	[11/15] 198468 - 198482
		384659 - 403461	18803	−	6	384659 - 384758 Len: 100	403361 - 403461 Len: 101	[14/17] 403287 - 403303 (-GlyTCC)	[13/15] 384769 - 384783
	1154.1	69587 - 87034	17448	−	6	69587 - 69692 Len: 106	86923 - 87034 Len: 112	[14/16] 86855 - 86870 (-GluTTC)	[12/15] 69733 - 69747
		197004 - 211504	14501	+	7	197004 - 197360 Len: 357	211156 - 211504 Len: 349	[16/17] 197410 - 197426 (LeuCAA)	[12/15] 211080 - 211094
	612.1	279163 - 287299	8137	−	6	279163 - 279320 Len: 158	287149 - 287299 Len: 151	[15/21] 287078 - 287098 (-GlnCTG)	[12/15] 279366 - 279380
	1817.1	132492 - 141135	8644	+	7	132492 - 132644 Len: 153	140982 - 141135 Len: 154	[15/21] 132713 - 132733 (LeuTAA)	[11/15] 140940 - 140954
	895.1	139108 - 146591	7484	−	6	139108 - 139287 Len: 180	146371 - 146591 Len: 221	[14/18] 146332 - 146349 (-PheGAA)	ND
	1080.1*	9 - 5995	5987	+	6	9 - 128 Len: 120	5879 - 5995 Len: 117	ND	[11/15] 5803 - 5817
		9 - 6856	6848	+	6	9 - 136 Len: 128	6725 - 6856 Len: 132	ND	[11/15] 6651 - 6665
		105049 - 106815	1767	−	6	105049 - 105166 Len: 118	106698 - 106815 Len: 118	[14/21] 106638 - 106658 (-SerCGA)	ND
		105049 - 109600	4552	+	6	105049 - 105166 Len: 118	109483 - 109600 Len: 118	ND	[11/15] 109391 - 109405
		105049 - 109887	4839	+	6	105049 - 105166 Len: 118	109770 - 109887 Len: 118	ND	[11/15] 109723 - 109737
		106135 - 109652	3518	+	6	106135 - 106327 Len: 193	109483 - 109652 Len: 170	ND	[11/15] 109391 - 109405
		106322 - 108000	1679	−	6	106322 - 106429 Len: 108	107889 - 108000 Len: 112	ND	[12/15] 106508 - 106522
		106363 - 108300	1938	−	6	106363 - 106523 Len: 161	108149 - 108300 Len: 152	ND	[11/15] 106600 - 106614
		106363 - 108455	2093	−	6	106363 - 106639 Len: 277	108149 - 108455 Len: 307	ND	[11/15] 106706 - 106720
	684.1	67758 - 75155	7398	+	6	67758 - 68154 Len: 397	74767 - 75155 Len: 389	[16/19] 68219 - 68237 (IleTAT)	[11/15] 74721 - 74735
		69889 - 76205	6317	−	6	69889 - 70124 Len: 236	75999 - 76205 Len: 207	[14/21] 75923 - 75943 (-GluCTC)	[12/15] 70205 - 70219
		69889 - 76435	6547	+	6	69889 - 70350 Len: 462	75999 - 76435 Len: 437	[16/27] 70388 - 70414 (ProTGG)	[11/15] 75936 - 75950
		69889 - 76599	6711	−	6	69889 - 70469 Len: 581	75999 - 76599 Len: 601	[14/21] 75923 - 75943 (-GluCTC)	[12/15] 70550 - 70564
		70134 - 76435	6302	+	6	70134 - 70350 Len: 217	76215 - 76435 Len: 221	[16/27] 70388 - 70414 (ProTGG)	[11/15] 76140 - 76154
		70134 - 76599	6466	−	6	70134 - 70469 Len: 336	76215 - 76599 Len: 385	[14/18] 76121 - 76138 (-GlnCTG)	[12/15] 70550 - 70564
		159518 - 160782	1265	−	6.5	159518 - 159635 Len: 118	160666 - 160782 Len: 117	[15/21] 160643 - 160663 (-LeuAAG)	[11/15] 159701 - 159715
		184999 - 187139	2141	−	6	184999 - 185274 Len: 276	186891 - 187139 Len: 249	[15/20] 186792 - 186811 (-ThrCGT)	[11/15] 185299 - 185313
		185026 - 187021	1996	−	6	185026 - 185466 Len: 441	186619 - 187021 Len: 403	ND	[14/15] 185481 - 185495
		241203 - 249130	7928	+	6	241203 - 241415 Len: 213	248918 - 249130 Len: 213	[15/19] 241430 - 241448 (PheGAA)	[13/15] 248898 - 248912
	228.1	275218 - 276629	1412	+	6	275218 - 275420 Len: 203	276458 - 276629 Len: 172	ND	[12/15] 276438 - 276452
		275429 - 277241	1813	−	6	275429 - 275615 Len: 187	277109 - 277241 Len: 133	ND	[11/15] 275661 - 275675
	357.1	62674 - 72364	9691	+	6	62674 - 63129 Len: 456	71911 - 72364 Len: 454	[14/19] 63207 - 63225 (ThrCGT)	[14/15] 71896 - 71910
		149759 - 161813	12055	+	7	149759 - 149917 Len: 159	161655 - 161813 Len: 159	[15/21] 149921 - 149941 (ValCAC)	[12/15] 161640 - 161654
		180238 - 182969	2732	+	6	180238 - 180551 Len: 314	182656 - 182969 Len: 314	ND	[11/15] 182587 - 182601
		210322 - 212492	2171	+	6	210322 - 210504 Len: 183	212265 - 212492 Len: 228	[15/20] 210555 - 210574 (MetCAT)	[12/15] 212213 - 212227
		210596 - 212731	2136	+	7	210596 - 210879 Len: 284	212473 - 212731 Len: 259	[14/20] 210882 - 210901 (SerTGA)	[12/15] 212383 - 212397
		210769 - 212731	1963	+	7	210769 - 210879 Len: 111	212620 - 212731 Len: 112	[14/20] 210882 - 210901 (SerTGA)	[12/15] 212581 - 212595
		210769 - 212908	2140	+	6	210769 - 211104 Len: 336	212620 - 212908 Len: 289	ND	[12/15] 212581 - 212595

	1344.1	111723 - 113537	1815	−	6	111723 - 111982 Len: 260	113295 - 113537 Len: 243	[14/18] 113207 - 113224 (-GlnCTG)	[14/15] 111996 - 112010
	1074.1	81236 - 82665	1430	−	6	81236 - 81459 Len: 224	82476 - 82665 Len: 190	[15/20] 82453 - 82472 (-LeuTAA)	[13/15] 81491 - 81505
		159010 - 160425	1416	−	6.5	159010 - 159205 Len: 196	160249 - 160425 Len: 177	[14/17] 160157 - 160173 (-IleAAT)	[11/15] 159221 - 159235
		159063 - 160349	1287	−	7	159063 - 159181 Len: 119	160249 - 160349 Len: 101	[14/17] 160157 - 160173 (-IleAAT)	[11/15] 159221 - 159235
		214814 - 217867	3054	−	6	214814 - 214921 Len: 108	217760 - 217867 Len: 108	ND	[11/15] 214922 - 214936
	193.1	143198 - 147027	3830	+	6	143198 - 143975 Len: 778	146229 - 147027 Len: 799	[14/19] 143979 - 143997 (GlnCTG)	[13/15] 146152 - 146166
		143278 - 146193	2916	+	6	143278 - 143505 Len: 228	145949 - 146193 Len: 245	[14/18] 143524 - 143541 (IleTAT)	[12/15] 145853 - 145867
		143676 - 147484	3809	+	6	143676 - 143807 Len: 132	147328 - 147484 Len: 157	[15/19] 143835 - 143853 (MetCAT)	[12/15] 147312 - 147326
		143813 - 147027	3215	+	6	143813 - 143975 Len: 163	146866 - 147027 Len: 162	[14/19] 143979 - 143997 (GlnCTG)	[11/15] 146778 - 146792
		189084 - 191040	1957	−	7	189084 - 189277 Len: 194	190843 - 191040 Len: 198	[16/21] 190754 - 190774 (-IleAAT)	[11/15] 189285 - 189299
	24.1	242805 - 244398	1594	+	6	242805 - 243036 Len: 232	244152 - 244398 Len: 247	ND	[12/15] 244107 - 244121
		287257 - 289315	2059	+	6	287257 - 287531 Len: 275	289026 - 289315 Len: 290	ND	[11/15] 288963 - 288977
		287298 - 289180	1883	−	6	287298 - 287420 Len: 123	289064 - 289180 Len: 117	ND	[12/15] 287447 - 287461
		460580 - 468901	8322	+	6	460580 - 460755 Len: 176	468725 - 468901 Len: 177	ND	[13/15] 468630 - 468644
	429.1	89475 - 95162	5688	−	6	89475 - 89620 Len: 146	95018 - 95162 Len: 145	ND	[13/15] 89688 - 89702
	2692.1	62963 - 64872	1910	+	6	62963 - 63180 Len: 218	64634 - 64872 Len: 239	ND	[12/15] 64594 - 64608
	893.1	2955 - 10919	7965	−	6.5	2955 - 3075 Len: 121	10800 - 10919 Len: 120	[14/20] 10725 - 10744 (-SerTGA)	[12/15] 3089 - 3103
		75145 - 83093	7949	+	6	75145 - 75279 Len: 135	82960 - 83093 Len: 134	[15/21] 75290 - 75310 (GlnTTG)	[13/15] 82877 - 82891
		85020 - 86568	1549	+	6	85020 - 85166 Len: 147	86423 - 86568 Len: 146	[14/20] 85192 - 85211 (PheGAA)	[13/15] 86324 - 86338
		85020 - 87877	2858	+	6	85020 - 85204 Len: 185	87692 - 87877 Len: 186	[14/18] 85277 - 85294 (SerCGA)	[14/15] 87609 - 87623
		85132 - 88028	2897	+	6	85132 - 85268 Len: 137	87891 - 88028 Len: 138	[14/18] 85277 - 85294 (SerCGA)	[11/15] 87848 - 87862
		85192 - 89954	4763	−	6	85192 - 85938 Len: 747	89254 - 89954 Len: 701	[15/19] 89156 - 89174 (-AlaTGC)	[11/15] 85957 - 85971
		85323 - 89954	4632	+	6	85323 - 85938 Len: 616	89370 - 89954 Len: 585	[14/19] 85987 - 86005 (ProTGG)	[11/15] 89355 - 89369
		85323 - 89980	4658	+	6	85323 - 85964 Len: 642	89370 - 89980 Len: 611	[14/19] 85987 - 86005 (ProTGG)	[11/15] 89355 - 89369
		85796 - 89980	4185	+	6	85796 - 85964 Len: 169	89853 - 89980 Len: 128	[14/19] 85987 - 86005 (ProTGG)	[11/15] 89838 - 89852
		85903 - 87446	1544	+	6	85903 - 86100 Len: 198	87249 - 87446 Len: 198	[15/22] 86155 - 86176 (GlnCTG)	[12/15] 87177 - 87191
		87951 - 89391	1441	−	6	87951 - 88088 Len: 138	89254 - 89391 Len: 138	[15/19] 89156 - 89174 (-AlaTGC)	[12/15] 88111 - 88125
	118.1	14575 - 16472	1898	−	6	14575 - 14758 Len: 184	16286 - 16472 Len: 187	[14/18] 16262 - 16279 (-ArgTCT)	[11/15] 14811 - 14825
		14575 - 16685	2111	−	6	14575 - 14925 Len: 351	16286 - 16685 Len: 400	[14/18] 16262 - 16279 (-ArgTCT)	[11/15] 14959 - 14973
		18699 - 21393	2695	−	6	18699 - 19035 Len: 337	21041 - 21393 Len: 353	ND	[11/15] 19036 - 19050
		69834 - 71178	1345	+	6	69834 - 69996 Len: 163	71021 - 71178 Len: 158	ND	[11/15] 70972 - 70986
		76336 - 77823	1488	+	7	76336 - 76493 Len: 158	77668 - 77823 Len: 156	[14/19] 76575 - 76593 (MetCAT)	[12/15] 77594 - 77608
		76336 - 78568	2233	+	7	76336 - 76493 Len: 158	78414 - 78568 Len: 155	[14/19] 76575 - 76593 (MetCAT)	[12/15] 78390 - 78404
		216947 - 236907	19961	+	6	216947 - 217171 Len: 225	236693 - 236907 Len: 215	[14/19] 217179 - 217197 (IleTAT)	[12/15] 236660 - 236674
		402912 - 404459	1548	+	6	402912 - 403012 Len: 101	404359 - 404459 Len: 101	ND	[12/15] 404306 - 404320
		533681 - 535037	1357	−	6	533681 - 533828 Len: 148	534889 - 535037 Len: 149	ND	[14/15] 533888 - 533902

		533681 - 535050	1370	−	6	533681 - 533866 Len: 186	534889 - 535050 Len: 162	ND	[14/15] 533888 - 533902
	1269.1	63723 - 72250	8528	−	7	63723 - 63869 Len: 147	72104 - 72250 Len: 147	[15/21] 72059 - 72079 (-SerAGA)	[13/15] 63882 - 63896
	1989.1	138962 - 140373	1412	+	6	138962 - 139106 Len: 145	140238 - 140373 Len: 136	[15/19] 139110 - 139128 (SerTGA)	[12/15] 140204 - 140218
	202.1	330201 - 338192	7992	−	6	330201 - 330435 Len: 235	337955 - 338192 Len: 238	[14/20] 337863 - 337882 (-ThrCGT)	[12/15] 330483 - 330497
		331030 - 333855	2826	−	6.5	331030 - 331345 Len: 316	333535 - 333855 Len: 321	[15/21] 333490 - 333510 (-GlnCTG)	[12/15] 331413 - 331427
		335519 - 337527	2009	+	6	335519 - 335941 Len: 423	337092 - 337527 Len: 436	[14/17] 336008 - 336024 (LeuCAA)	[13/15] 337077 - 337091
		418240 - 420681	2442	−	6	418240 - 418807 Len: 568	420102 - 420681 Len: 580	[14/20] 420009 - 420028 (-GluCTC)	[13/15] 418833 - 418847

a: Score is an integer varying from 0 to 11.

b & c: Location of the LTRs. 5'LTR and 3'LTR are two similar regions. A typical LTR retrotransposon has a structure called TG..CA box, with TG at the 5' extremity of 5'LTR and CA at the 3' extremity of 3'LTR.

d: The first number in square brackets is number of matched bases and the second is total alignment length. Following is signal positions. String in parentheses is the tRNA type and anti-codon.

e: The first is the number of purines and length of putative PPT.

ND: Not detected.

*: Only first 9 outputs are shown out of 24

Supplementary Table S7: Features of LTR-retrotransposons identified with NRPS-associated scaffolds studied in this paper.

Clade	Scaffold #	Location	Length	Strand	Score[a]	5'-LTR[b]	3'-LTR[c]	PBS (Primer Binding Sites)[d]	PPT (Polypurine tract)[e]
A3	451.1	124432 - 127126	2695	+	6	124432 - 124613 Len: 182	126946 - 127126 Len: 181	[15/20] 124630 - 124649 (GlnCTG)	[13/15] 126920 - 126934
		124768 - 129338	4571	+	6	124768 - 124899 Len: 132	129207 - 129338 Len: 132	[14/20] 124958 - 124977 (AlaTGC)	[12/15] 129110 - 129124
	112.1	130245 - 133532	3288	+	6	130245 - 130356 Len: 112	133421 - 133532 Len: 112	[14/20] 130365 - 130384 (PheGAA)	[12/15] 133404 - 133418
		130357 - 133527	3171	−	6	130357 - 130471 Len: 115	133408 - 133527 Len: 120	ND	[11/15] 130549 - 130563
	1977.1	36433 - 43341	6909	−	6.5	36433 - 36594 Len: 162	43179 - 43341 Len: 163	[16/24] 43125 - 43148 (-AsnGTT)	[14/15] 36633 - 36647
		122602 - 124406	1805	+	6	122602 - 122819 Len: 218	124212 - 124406 Len: 195	[14/19] 122867 - 122885 (LeuTAG)	[13/15] 124117 - 124131
	2138.1	6877 - 22893	16017	+	6	6877 - 6982 Len: 106	22788 - 22893 Len: 106	[15/19] 7025 - 7043 (LeuTAG)	[12/15] 22698 - 22712
		22900 - 27800	4901	+	6.5	22900 - 23005 Len: 106	27699 - 27800 Len: 102	[14/17] 23040 - 23056 (IleTAT)	[11/15] 27659 - 27673
	745.1	22201 - 23780	1580	−	6	22201 - 22314 Len: 114	23668 - 23780 Len: 113	[15/22] 23584 - 23605 (-IleAAT)	[11/15] 22396 - 22410
B1	3629.1	16587 - 24405	7819	−	7	16587 - 16755 Len: 169	24237 - 24405 Len: 169	ND	[12/15] 16756 - 16770
	4570.1	19443 - 27781	8339	−	7	19443 - 19722 Len: 280	27502 - 27781 Len: 280	ND	[12/15] 19768 - 19782
C	535.1	288896 - 297474	8579	+	6.5	288896 - 288996 Len: 101	297372 - 297474 Len: 103	[14/19] 289012 - 289030 (MetCAT)	[12/15] 297357 - 297371
	1214.1	142212 - 157440	15229	−	6	142212 - 142355 Len: 144	157290 - 157440 Len: 151	[15/22] 157211 - 157232 (-AlaCGC)	[13/15] 142356 - 142370
	193.1	143198 - 147027	3830	+	6	143198 - 143975 Len: 778	146229 - 147027 Len: 799	[14/19] 143979 - 143997 (GlnCTG)	[13/15] 146152 - 146166
		143278 - 146193	2916	+	6	143278 - 143505 Len: 228	145949 - 146193 Len: 245	[14/18] 143524 - 143541 (IleTAT)	[12/15] 145853 - 145867
		143676 - 147484	3809	+	6	143676 - 143807 Len: 132	147328 - 147484 Len: 157	[15/19] 143835 - 143853 (MetCAT)	[12/15] 147312 - 147326
		143813 - 147027	3215	+	6	143813 - 143975 Len: 163	146866 - 147027 Len: 162	[14/19] 143979 - 143997 (GlnCTG)	[11/15] 146778 - 146792
		189084 - 191040	1957	−	7	189084 - 189277 Len: 194	190843 - 191040 Len: 198	[16/21] 190754 - 190774 (-IleAAT)	[11/15] 189285 - 189299
	2146.1	31479 - 36824	5346	−	7	31479 - 31731 Len: 253	36571 - 36824 Len: 254	[14/19] 36547 - 36565 (-ProTGG)	[12/15] 31761 - 31775
		32468 - 37643	5176	−	6	32468 - 32971 Len: 504	37158 - 37643 Len: 486	[14/22] 37077 - 37098 (-AlaAGC)	[12/15] 33010 - 33024
		35948 - 40886	4939	−	6	35948 - 36131 Len: 184	40707 - 40886 Len: 180	[14/20] 40631 - 40650 (-LeuTAA)	ND
		36018 - 41388	5371	+	7	36018 - 36539 Len: 522	40877 - 41388 Len: 512	[15/21] 36608 - 36628 (SerTGA)	[13/15] 40794 - 40808
	227.1*	5759 - 37699	1941	+	6	35759 - 36172 Len: 414	37285 - 37699 Len: 415	[15/19] 36253 - 36271 (LeuCAA)	[11/15] 37226 - 37240
		85652 - 91173	5522	−	6	85652 - 86139 Len: 488	90728 - 91173 Len: 446	[14/19] 90701 - 90719 (-AlaTGC)	[11/15] 86156 - 86170
		85652 - 91219	5568	−	6	85652 - 86139 Len: 488	90728 - 91219 Len: 492	[14/19] 90701 - 90719 (-AlaTGC)	[11/15] 86156 - 86170
		85652 - 91265	5614	−	6	85652 - 86093 Len: 442	90772 - 91265 Len: 494	[14/19] 90701 - 90719 (-AlaTGC)	[11/15] 86156 - 86170
		85652 - 91276	5625	+	6	85652 - 86075 Len: 424	90864 - 91276 Len: 413	[14/19] 86093 - 86111 (SerCGA)	[11/15] 90781 - 90795

		85652 - 91401	5750	+	6	85652 - 85970 Len: 319	91102 - 91401 Len: 300	ND	[11/15] 91019 - 91033
		85699 - 91081	5383	−	6	85699 - 86139 Len: 441	90682 - 91081 Len: 400	[15/23] 90651 - 90673 (-IleAAT)	[11/15] 86156 - 86170
		85745 - 91127	5383	−	6	85745 - 86139 Len: 395	90682 - 91127 Len: 446	[15/23] 90651 - 90673 (-IleAAT)	[11/15] 86156 - 86170
		85791 - 91035	5245	−	6	85791 - 86139 Len: 349	90682 - 91035 Len: 354	[15/23] 90651 - 90673 (-IleAAT)	[11/15] 86156 - 86170
	268.1	334018 - 342657	8640	+	8	334018 - 334147 Len: 130	342526 - 342657 Len: 132	[16/23] 334160 - 334182 (GlnCTG)	[11/15] 342478 - 342492
	830.1	42518 - 44223	1706	−	6	42518 - 42693 Len: 176	44044 - 44223 Len: 180	[14/18] 43968 - 43985 (-IleAAT)	[11/15] 42695 - 42709
		120620 - 122987	2368	+	6	120620 - 120849 Len: 230	122781 - 122987 Len: 207	[14/19] 120862 - 120880 (IleAAT)	[11/15] 122722 - 122736
		120652 - 123784	3133	+	6	120652 - 120868 Len: 217	123569 - 123784 Len: 216	[14/18] 120938 - 120955 (LeuTAG)	[11/15] 123527 - 123541
		120899 - 123577	2679	+	7	120899 - 121301 Len: 403	123213 - 123577 Len: 365	[14/18] 121338 - 121355 (LeuTAG)	[11/15] 123176 - 123190
		120899 - 123577	2679	+	7	120899 - 121166 Len: 268	123349 - 123577 Len: 229	[14/18] 121210 - 121227 (LeuTAG)	[11/15] 123255 - 123269
		120899 - 123577	2679	+	7	120899 - 121429 Len: 531	123077 - 123577 Len: 501	[14/18] 121471 - 121488 (LeuTAG)	[11/15] 123040 - 123054
		121125 - 123024	1900	+	6	121125 - 121515 Len: 391	122653 - 123024 Len: 372	ND	[11/15] 122585 - 122599

a: Score is an integer varying from 0 to 11.

b & c: Location of the LTRs. 5'LTR and 3'LTR are two similar regions. A typical LTR retrotransposon has a structure called TG..CA box, with TG at the 5' extremity of 5'LTR and CA at the 3' extremity of 3'LTR.

d: The first number in square brackets is number of matched bases and the second is total alignment length.Following is signal positions. String in parentheses is the tRNA type and anti-codon.

e: The first is the number of purines and length of putative PPT.

ND: Not detected.

*: Only first 9 outputs are shown out of 24

Supplementary Table S8: Details of NCBI, MMETSP and *Symbiodinium kawagutii* sequences belonging to the Dinoflagellate PKS clade (Green insert) in Fig. 1

**NCBI**	
**Sequence name**	**Accession Number/ gene ID**
*A.spinosum*_AS3D902	AIW63286.1
*K.brevis*_KB5361	ABQ85800.1
*G.polynesiensis*_AHM27264.1	AHM27264.1
*A.spinosum*_AS3D903	AIW63287.1
*A.ostenfeldii*_AFW98413.1	AFW98413.1
*K.brevis*_KB2006	ABQ85797.1
*A.ostenfeldii*_AFW98412.1	AFW98412.1
*A.spinosum*_AS3D904	AIW63288.1
*H.triquetra*_AFW98415.1	AFW98415.1
*S.kawagutii*_222406	Skav222406
*K.brevis*_KB4825	ABQ85798.1
*K.brevis*_KB1008	ABQ85796.1

**MMETSP**	
**Sequence name**	**Sequence ID**
*Symbiodinium*_A_KS0042291333	CAMPEP_0196899616 /NCGR_PEP_ID=MMETSP1374-20130617|79528_1
*Symbiodinium*_A_KS0042212549	CAMPEP_0196831228 /NCGR_PEP_ID=MMETSP1374-20130617|1885_1
*Symbiodinium*_D1a_KS 0042383695	CAMPEP_0196972188 /NCGR_PEP_ID=MMETSP1377-20130617|114813_1
*Symbiodinium*_A_KS0042213687	CAMPEP_0196832172 /NCGR_PEP_ID=MMETSP1374-20130617|2503_1
*Symbiodinium*_D1a_ KS0042406815	CAMPEP_0196991650 /NCGR_PEP_ID=MMETSP1377-20130617|138647_1
*Symbiodinium*_A_KS0042228871	CAMPEP_0196845784 /NCGR_PEP_ID=MMETSP1374-20130617|10937_1
*Symbiodinium*_D1a_0042350567	CAMPEP_0196945412 /NCGR_PEP_ID=MMETSP1377-20130617|71860_1
*Symbiodinium*_C1_KS0045394493	CAMPEP_0199555398 /NCGR_PEP_ID=Symbiodinium-sp-C1-20140214|916_1
*Symbiodinium*_C1_KS0045406663	CAMPEP_0199566116 /NCGR_PEP_ID=Symbiodinium-sp-C1-20140214|7881_1
*Symbiodinium*_sp-Mp_KS0036770103	CAMPEP_0192565212 /NCGR_PEP_ID=Symbiodinium-sp-Mp-20130822|31_1
Symbiodinium _C1a_KS0045417207	CAMPEP_0199575736 /NCGR_PEP_ID=Symbiodinium-sp-C1-20140214|13569_1
*Symbiodinium*_sp-Mp_KS0036784927	CAMPEP_0192578502 /NCGR_PEP_ID=Symbiodinium-sp-Mp-20130822|8950_1
*Symbiodinium*_C1_KS0045402761	CAMPEP_0199562872 /NCGR_PEP_ID=Symbiodinium-sp-C1-20140214|5679_1
*Symbiodinium*_C1a_KS0045408335	CAMPEP_0199567626 /NCGR_PEP_ID=Symbiodinium-sp-C1-20140214|8745_1
*Symbiodinium*_D1a_KS0042408223	CAMPEP_0196993016 /NCGR_PEP_ID=MMETSP1377-20130617|139351_1
*Symbiodinium*_sp-Mp_KS0036814021	CAMPEP_0192602342 /NCGR_PEP_ID=Symbiodinium-sp-Mp-20130822|27721_1
*Symbiodinium*_A_ KS0042218263	CAMPEP_0196836150 /NCGR_PEP_ID=MMETSP1374-20130617|4963_1
*Symbiodinium*_D1a KS0042389817	CAMPEP_0196977200 /NCGR_PEP_ID=MMETSP1377-20130617|122114_1
*Symbiodinium*_C1_KS0045399549	CAMPEP_0199560036 /NCGR_PEP_ID=Symbiodinium-sp-C1-20140214|3696_1
*A.carterae*_KS0029207815	CAMPEP_0186409720 /NCGR_PEP_ID=Amphidinium-carterae-CCMP1314-20130924|551_1
*A.carterae*_KS0029210987	CAMPEP_0186412490 /NCGR_PEP_ID=Amphidinium-carterae-CCMP1314-20130924|2444_1
*A.carterea*_KS0029207525	CAMPEP_0186409468 /NCGR_PEP_ID=Amphidinium-carterae-CCMP1314-20130924|381_1

*A.carterae*_KS0029266881	CAMPEP_0186460252 /NCGR_PEP_ID=Amphidinium-carterae-CCMP1314-20130924|62901_1
*A.carterae*_KS0029214519	CAMPEP_0186415646 /NCGR_PEP_ID=Amphidinium-carterae-CCMP1314-20130924|4682_1
*P.reticulatum*_KS0008371577	CAMPEP_0168358974 /NCGR_PEP_ID=MMETSP0228-20121227|1397_1
*P.reticulatum*_KS0008381755	CAMPEP_0168368902 /NCGR_PEP_ID=MMETSP0228-20121227|6486_1
*P.beii*_KS0043195877	CAMPEP_0197625458 /NCGR_PEP_ID=MMETSP1338-20131121|4820_1
*P.beii*_KS0043213389	CAMPEP_0197640526 /NCGR_PEP_ID=MMETSP1338-20131121|14787_1
P.beii KS0043208619	CAMPEP_0197636402 /NCGR_PEP_ID=MMETSP1338-20131121|11914_1
*G.catenatum*_KS0005298767	CAMPEP_0117503450 /NCGR_PEP_ID=MMETSP0784-20121206|24336_1
*G.catenatum*_KS0005265677	CAMPEP_0117471558 /NCGR_PEP_ID=MMETSP0784-20121206|7791_1
*P.bahamense*_KS0020712219	CAMPEP_0179016528 /NCGR_PEP_ID=MMETSP0796-20121207|3365_1
*P.bahamense*_KS0020707261	CAMPEP_0179011850 /NCGR_PEP_ID=MMETSP0796-20121207|886_1
